# Digital Action Plan (Web App) for Managing Asthma Exacerbations: Randomized Controlled Trial

**DOI:** 10.2196/41490

**Published:** 2023-06-29

**Authors:** Nicole Beydon, Camille Taillé, Harriet Corvol, Judith Valcke, Jean-Jacques Portal, Laurent Plantier, Gilles Mangiapan, Caroline Perisson, Guillaume Aubertin, Alice Hadchouel, Guillaume Briend, Laurent Guilleminault, Catherine Neukirch, Pierrick Cros, Corinne Appere de Vecchi, Bruno Mahut, Eric Vicaut, Christophe Delclaux

**Affiliations:** 1 Unité Fonctionnelle de Physiologie-Explorations Fonctionnelles Respiratoires, Institut National de la Santé et de la Recherche Médicale 938, Centre de Recherche Saint Antoine, Hôpital Armand Trousseau, Assistance Publique Hôpitaux de Paris, F-75012 Paris France; 2 Service de Pneumologie et Centre de Référence Constitutif des Maladies Pulmonaires Rares, Hôpital Bichat, Assistance Publique Hôpitaux de Paris, Institut National de la Santé et de la Recherche Médicale 1152, Université Paris Cité, F-75018 Paris France; 3 Service de Pneumologie Pédiatrique, Hôpital Armand Trousseau, Assistance Publique Hôpitaux de Paris, Institut National de la Santé et de la Recherche Médicale Centre de Recherche Saint-Antoine, Sorbonne Université, F-75012 Paris France; 4 Service de Pneumologie, Hôpital Européen Georges Pompidou, Assistance Publique Hôpitaux de Paris, F-75015 Paris Hôpital Privé Armand Brillard, F-94130 Paris France; 5 Clinical Research Unit Saint-Louis Lariboisière, Assistance Publique Hôpitaux de Paris, Université de Paris Cité, F-75010 Paris France; 6 Département de Pneumologie et Explorations Fonctionnelles Respiratoires, Centre Hospitalier Universitaire de Tours, Institut National de la Santé et de la Recherche Médicale unité 1100, Université de Tours, F-37000 Tours France; 7 Service de Pneumologie, Centre Hospitalier Interrégional de Créteil, F-94010 Créteil France; 8 Centre de pneumologie et d'allergologie de l'enfant, F-92100 Boulogne Billancourt France; 9 Service de Pneumologie Pédiatrique, Centre de Référence pour les Maladies Respiratoires Rares de l'Enfant, Hôpital Universitaire Necker-Enfants Malades, Assistance Publique Hôpitaux de Paris, Université de Paris Cité, F-75015 Paris France; 10 Service de Pneumologie, Centre hospitalier de Pontoise, F-95303 Cergy Pontoise France; 11 Département de Pneumologie et Allergologie, Centre Hospitalo-Universitaire Purpan, Centre National de la Recherche Scientifique U5282, Institut National de la Santé et de la Recherche Médicale U1291, Toulouse Institute for Infectious, Inflammatory Disease Toulouse France; 12 Service de Pneumologie et Centre de Référence Constitutif des Maladies Pulmonaires Rares, Hôpital Bichat, Assistance Publique des Hôpitaux de Paris, Institut National de la Santé et de la Recherche Médicale 1152, F-75018 Paris France; 13 Département de Pédiatrie, Hôpital Universitaire Morvan, F-29200 Brest France; 14 Service de Pneumologie, Centre Hospitalier Victor Dupouy, F-95100 Argenteuil France; 15 Cabinet La Berma, F-92160 Antony France; 16 Service de Physiologie Pédiatrique-Centre du Sommeil, Hôpital Robert Debré, Assistance Publique Hôpitaux de Paris, Institut National de la Santé et de la Recherche Médicale NeuroDiderot, Université de Paris Cité, F-75019 Paris France

**Keywords:** asthma exacerbation, action plan, web application, mobile phone

## Abstract

**Background:**

A written action plan (WAP) for managing asthma exacerbations is recommended.

**Objective:**

We aimed to compare the effect on unscheduled medical contacts (UMCs) of a digital action plan (DAP) accessed via a smartphone web app combined with a WAP on paper versus that of the same WAP alone.

**Methods:**

This randomized, unblinded, multicenter (offline recruitment in private offices and public hospitals), and parallel-group trial included children (aged 6-12 years) or adults (aged 18-60 years) with asthma who had experienced at least 1 severe exacerbation in the previous year. They were randomized to a WAP or DAP+WAP group in a 1:1 ratio. The DAP (fully automated) provided treatment advice according to the severity and previous pharmacotherapy of the exacerbation. The DAP was an algorithm that recorded 3 to 9 clinical descriptors. In the app, the participant first assessed the severity of their current symptoms on a 10-point scale and then entered the symptom descriptors. Before the trial, the wordings and ordering of these descriptors were validated by 50 parents of children with asthma and 50 adults with asthma; the app was not modified during the trial. Participants were interviewed at 3, 6, 9, and 12 months to record exacerbations, UMCs, and WAP and DAP use, including the subjective evaluation (availability and usefulness) of the action plans, by a research nurse.

**Results:**

Overall, 280 participants were randomized, of whom 33 (11.8%) were excluded because of the absence of follow-up data after randomization, leaving 247 (88.2%) participants (children: n=93, 37.7%; adults: n=154, 62.3%). The WAP group had 49.8% (123/247) of participants (children: n=45, 36.6%; mean age 8.3, SD 2.0 years; adults: n=78, 63.4%; mean age 36.3, SD 12.7 years), and the DAP+WAP group had 50.2% (124/247) of participants (children: n=48, 38.7%; mean age 9.0, SD 1.9 years; adults: n=76, 61.3%; mean age 34.5, SD 11.3 years). Overall, the annual severe exacerbation rate was 0.53 and not different between the 2 groups of participants. The mean number of UMCs per year was 0.31 (SD 0.62) in the WAP group and 0.37 (SD 0.82) in the DAP+WAP group (mean difference 0.06, 95% CI −0.12 to 0.24; *P*=.82). Use per patient with at least 1 moderate or severe exacerbation was higher for the WAP (33/65, 51% vs 15/63, 24% for the DAP; *P*=.002). Thus, participants were more likely to use the WAP than the DAP despite the nonsignificant difference between the action plans in the subjective evaluation. Median symptom severity of the self-evaluated exacerbation was 4 out of 10 and not significantly different from the symptom severity assessed by the app.

**Conclusions:**

The DAP was used less often than the WAP and did not decrease the number of UMCs compared with the WAP alone.

**Trial Registration:**

ClinicalTrials.gov NCT02869958; https://clinicaltrials.gov/ct2/show/NCT02869958

## Introduction

### Background

A key goal of asthma care is to empower patients (or parents) to take active and independent control of their condition. A tool used to achieve this goal is a personalized asthma action plan consisting of self-management instructions devised during discussions with the patient [1,2]. To help maintain asthma control and regain control in the event of an exacerbation, the instructions indicate how to respond to worsening symptoms and when to seek medical help. Asthma guidelines recommend that patients receive a written action plan (WAP) on paper indicating how and when to take rescue medications, with the goals of supporting self-management and decreasing unscheduled medical contacts (UMCs) [1]. Nevertheless, WAPs are underused, and whether they decrease UMCs compared with asthma education alone is debated [3-6]. The WAP may not be on hand when symptoms occur and, given the wide variability in symptom severity, may fail to indicate the most appropriate course of action for each specific situation. Of the children observed for asthma at any of 6 French pediatric emergency departments, 38% could have been successfully treated at home [7]. Having a WAP at home was not independently associated with avoidable visits, for which the only independent risk factor was fear or anxiety in the patients and family [7]. Conceivably, digital action plans (DAPs) accessible via a smartphone might help promote successful self-management [8]. Many people have their smartphone with them at all times, and a DAP allows back-and-forth interaction with an algorithm, possibly providing better reassurance than instructions on paper. In 2012, 103 English-language smartphone apps for asthma were available, including 56 apps that provided only information on asthma in general and 47 apps that provided information on asthma management [9]. Previous randomized controlled trials of mobile phone apps for asthma self-monitoring focused on asthma control [10-14]. None provided detailed instructions on the use of rescue medications for the self-management of acute asthma exacerbations with the goal of decreasing UMCs. The DeLone and McLean Information Systems Success Model suggests that both intent to use and satisfaction depend on the quality of the information, system, and service [15]. High-quality information on asthma is readily available, and the quality of internet sites and apps has improved considerably. In a survey, patients with asthma preferred apps that provided action plans for handling exacerbations over those that provided only information [16]. Another study showed that patients considered that internet-of-things systems could usefully provide integrated support for a number of recognized components of self-management [17]. Finally, a recent exploratory sequential mixed methods study showed that patients expressed mostly wanting a system to log their asthma control status automatically, provide real-time advice to help them learn about their asthma, and adjust their treatment [18]. Moreover, access to clinical advice provided a feeling of safety to patients [18].

### This Study

We performed a randomized, unblinded, multicenter, and parallel-group trial in older children and adults with asthma to test the hypothesis that compared with a WAP alone, a DAP combined with a WAP would decrease UMCs for acute asthma exacerbations. The app allowed the patient or parent to obtain an assessment of exacerbation severity and indicated the course of action for each severity level, which had not previously been done. The secondary objectives were to compare the frequency of use between the DAP and WAP and to assess satisfaction with the DAP.

## Methods

### Study Design

**Figure 1:**
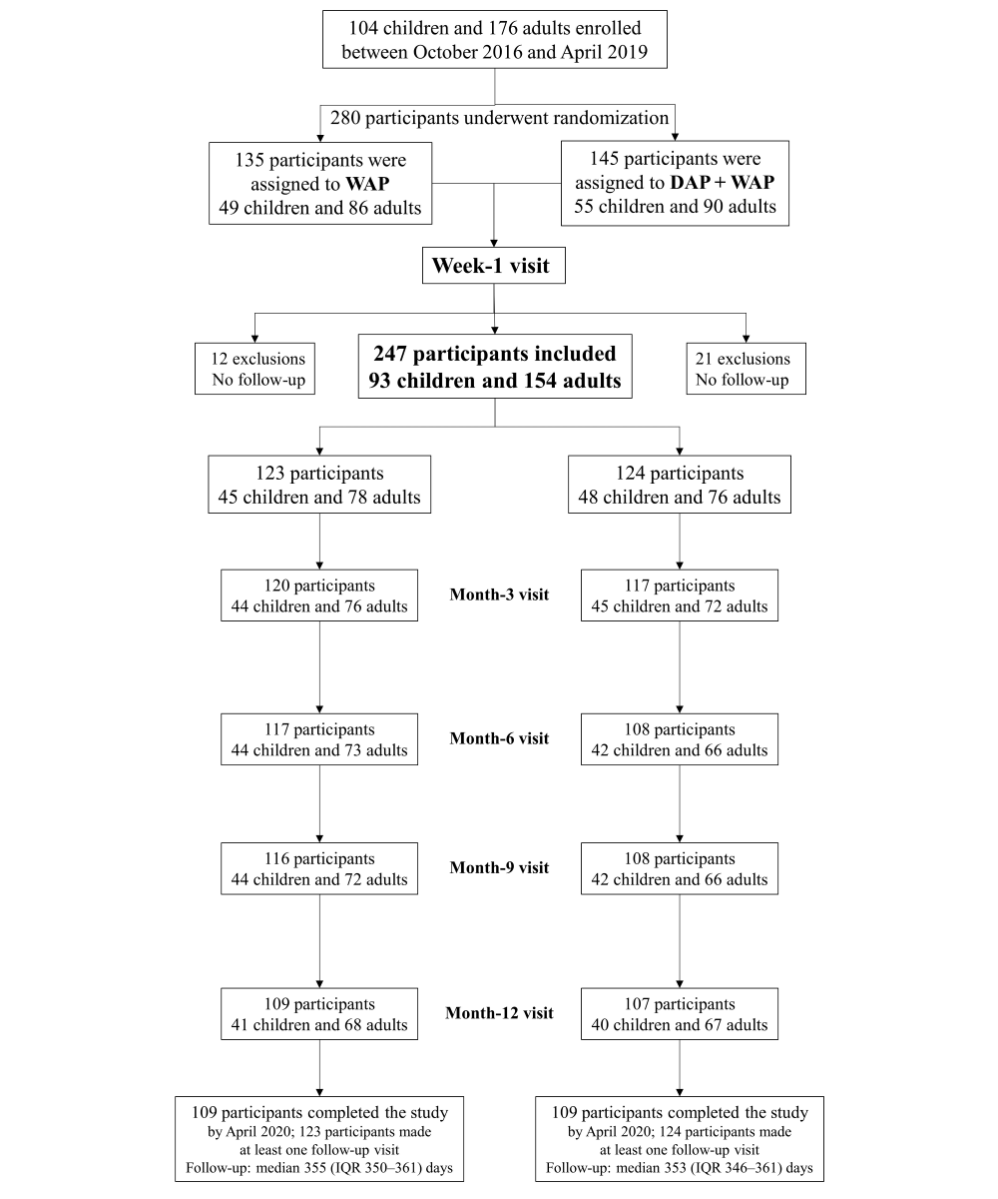
Participant flow chart WAP: written action plan; DAP: digital action plan.

This randomized, unblinded, multicenter, and parallel-group trial was performed in 27 French private offices and public hospitals (Figure 1).

### Ethics Approval, Informed Consent, and Participation

This study was approved by the appropriate ethics committee (*Comité de Protection des Personnes Ile de France 1*; #2016 janvier-14122-ND). Written informed consent was obtained from adult participants and from the guardians of pediatric participants. Study data were anonymous (data of the web app) or deidentified (data obtained in phone interviews). The participants received no financial compensation for their participation.

### Recruitment

Children aged 6 to 12 years and adults aged 18 to 60 years were eligible if they had a clinical diagnosis of asthma with at least 1 severe exacerbation requiring at least 3 days of systemic glucocorticoid therapy in the previous 12 months. Not having an action plan in the past year was an inclusion criterion. The ability to establish an internet connection through one’s own or a parent’s smartphone or tablet was also an inclusion criterion. Informed written consent for study participation involving several telephone or email interactions during the 1-year follow-up was obtained from each participant or parent. In accordance with the French law, we included only patients registered with the French statutory health insurance system.

Noninclusion criteria were atypical asthma (isolated cough or respiratory discomfort upon exercising), other respiratory diseases (eg, cystic fibrosis or chronic obstructive lung disease), severe cardiovascular disease other than hypertension, smoking history greater than 15 pack-years, and need for daily oral corticosteroid therapy or home nebulization therapy for asthma control. In addition, neither children with a sibling included in the study nor women who were pregnant or breastfeeding were eligible for the trial.

The only exclusion criterion was the absence of follow-up data after randomization.

Patients were recruited at hospitals or by office-based pediatricians and pulmonologists between October 2016 and April 2019.

### DAP Development

The DAP was developed by BM (Mosquitom Corporation, now dissolved). The participants had access to the DAP via a smartphone web app. The DAP was an algorithm that recorded 3 to 9 clinical descriptors; assessed whether they all belonged to the same severity level (mild, moderate, or severe); and provided advice about rescue medications to be taken over the next few hours and days based on severity, asthma controller treatment, and rescue medications already taken. The algorithm was not specifically designed for asthma exacerbations and was the property of Mosquitom (limitation for replicability); the algorithm was designed to weight the descriptor, as usefulness varies across descriptors in clinical practice. However, the clinical descriptors and appropriateness of the therapeutic advice were reviewed and accepted by 2 working groups led by representatives of the 2 French pulmonology societies for children and adults (*Société Pédiatrique de Pneumologie et d’Allergologie* and *Société de Pneumologie de Langue Française*, respectively).

The first step in DAP development was to define 9 descriptors of common manifestations of asthma exacerbations. To ensure that each descriptor would be easily understood by patients and families, we asked 50 parents of children with asthma and 50 adults with asthma to choose the best of 3 different wordings for each descriptor (involvement of participants in the design of the app; the contextual adaptability is thus limited to the French language).

The descriptors were then listed in the order in which they are usually recognized by patients and parents. The descriptors were those listed in the Global Initiative for Asthma (GINA), namely, breathlessness, comfortable posture (answer was mandatory), ability to talk (answer was mandatory), alertness, accessory muscle retractions, suprasternal retractions (or the use of accessory respiratory muscles), wheezing (audible), respiratory rate, and pulse rate (adults) or cough (children). Each descriptor was weighted according to its severity level (mild, moderate, or severe; Table 1).

To determine whether the severity levels defined for each descriptor were consistent with the severity categories in the GINA (mild, moderate, or severe), we obtained descriptions of 50 exacerbations in adults. We found no instances of inconsistencies, such as the same patient being comfortable lying down but being able to say only separate words.

To provide treatment advice, the app required at least 3 descriptors (of which 2 were mandatory), and the algorithm further determined whether additional descriptors were required. Once 3 descriptors had been entered, the app indicated its ability to give advice, but the patient was free to add other descriptors up to a total of 9 descriptors. If inconsistencies between the 9 descriptors remained, the app indicated its inability to provide advice. The app was “frozen” before the trial (no modification of the app during the trial).

**Table 1:** Symptoms recorded in the digital action plan.

Descriptors	Mild symptoms	Moderate symptoms	Severe symptoms
Breathlessness	While walking	While talking	At rest
Comfortable posture	Can lie down	Prefers sitting to lying	Sits hunched
Ability to talk	Conversation	Phrases	Words
Alertness and behavior	Not agitated	Agitated or anxious	Cannot move
Neck and chest muscle contraction or retractions	Absent	Mild	Frank
Wheezing^a^	Not or faintly audible	Audible	Strongly audible
Respiratory rate	Normal	Slightly increased	Greatly increased
Pulse rate (adult)	Normal	Slightly increased	Greatly increased
Nails and lips	Normal	Pallor	Cyanosis
Cough (child)	Slight	Persistent	Cannot breathe between coughs

^a^Wheezing audible to the patient or those near the patient. As assessing pulse rate normality in their children is difficult for parents, this descriptor was replaced by cough, which is common.

### A Posteriori Evaluation of the App Using the Guidelines for Reporting of Health Interventions Using Mobile Phones

We evaluated whether our app complied with the guidelines for reporting of health interventions using mobile phones [19]. These guidelines describe 16 items, including infrastructure (*Results* section), the technology platform (Multimedia Appendices 1 and 2), the intervention delivery (*Results* section), the intervention content (*Methods* section), usability and content testing (*Results* section), user feedback (*Results* section), adoption inputs and program entry (*Methods* section), limitations for delivery at scale (*Results* section), contextual adaptability (*Methods* section), replicability (*Methods* section), data security (*Methods* section), compliance with national guidelines or regulatory statutes (*Methods* section), and the fidelity of the intervention (*Methods* section). Two items of these guidelines (items 3 and 9) were not relevant to our study: interoperability and health information systems context and cost assessment. The social barriers to or facilitators of the adoption of the intervention among study participants were not evaluated (item 8 of the guidelines). Overall, our app complied with 13 of the 16 items.

### Adequacy of the Therapeutic Advice Provided by the DAP

At each connection of a participant for an exacerbation, the 2 asthma specialists NB and CD received an email describing the symptoms and advice provided by the app (backend data to track message delivery, the fidelity of the intervention, and data security). They then checked whether the advice was appropriate to the symptoms. Information was not shared with the physician in charge of the participant.

### Interventions

We compared a paper WAP to the same WAP combined with a DAP. The WAP for children was developed by a French pediatric pulmonology society (*Groupe de Recherche sur les Avancées en Pneumologie Pédiatrique*) [20], and the WAP for adults was developed by the GINA (2016) [1]. Both WAPs assessed severity based only on symptoms; thus, peak flow measurement was not required.

When using the app, the participant first assessed the severity of their current symptoms on a 10-point scale (0=no exacerbation; 10=maximum severity) and then entered the symptom descriptors. Once at least 3 of the 9 descriptors had been entered, the app assessed descriptor coherence and determined severity and then indicated which medications should be taken immediately and in the next few days and whether a physician visit was required.

The recruiting physician explained the WAP (there was no standardization of this asthma education session) and provided brief information about the DAP to each patient or family at inclusion. Each patient randomized to the DAP group was assigned an anonymized inclusion code for use in the DAP. Maintenance and rescue treatments prescribed by the physician were recorded in the app using the same names as those in the prescription (brand or generic). During a telephone interview (week-1 telephone interview in Figure 1), the research nurse helped the patient or parent establish their first connection to the app by entering data as if an exacerbation were occurring (training session); this validated app registration. The participants who did not attend this first telephone visit were excluded. The research nurses of the clinical research unit conducted follow-up interviews by telephone or email at 3, 6, 9, and 12 months. Asthma follow-up was at the discretion of the physician in charge of the patient. Information was not shared between the app and physicians.

Multimedia Appendix 1 provides demo pages of the trial: recording of the participant by the nurse, first connection of the participant (validation), and recording of an asthma attack.

### Randomization and Masking

The nurse practitioner of our clinical research unit randomized the patients in a 1:1 ratio to the WAP group or DAP+WAP group within 1 week after inclusion by connecting to a centralized web-based system. Randomization was performed in permuted blocks of randomly varying sizes with stratification based on age (children vs adults), recruitment site (hospital vs office), and the GINA asthma control score during the previous month. Participant blinding was not feasible. Group assignment was known to the participants and the clinical research unit but not to the trial investigators until the next follow-up visit with the patient.

Asthma control was categorized as reflecting controlled, partially controlled, or uncontrolled asthma (score of 0, 1 or 2, or 3 or 4, respectively) as proposed by the GINA [1].

### Outcomes

The primary outcome was the number of UMCs for asthma during the 1-year follow-up. UMCs included in-person physician and emergency department visits and remote contacts with health care workers by phone, SMS text message, or email.

The secondary outcomes were the proportion of reported exacerbations that were entered into the app, proportion of reported exacerbations for which participants reported using the WAP, subjective evaluation of the WAP and DAP by the participants at the end of the follow-up using a 1-10 visual analog scale (VAS), and GINA asthma control scores [1] reported during the telephone interviews at 3-month intervals during the 1-year follow-up.

All acute symptoms were to be reported by the participants during the telephone interviews. The research nurse would then categorize the occurrence of acute symptoms according to the American Thoracic Society-European Respiratory Society statement as mild (transient loss of control responding to single-day beta-agonist therapy), moderate (rescue beta-agonist therapy for at least 2 days), or severe exacerbation (oral glucocorticoid for at least 3 days in addition to beta-agonist therapy) [21].

### Statistical Analysis

#### Sample Size Estimation

On the basis of earlier studies, we assumed that approximately 80% of the participants would experience at least 1 moderate or severe exacerbation during the 1-year study period [22,23]. With the chi-square test and the 2-sided α risk set at 5%, 242 participants (121 in each group) were required to obtain at least 80% power for detecting a 20% between-group difference (relative effect). Previous studies suggested a 25% decrease in unscheduled health care contacts for severe exacerbations when a WAP is supplied [4,5]. However, we expected that adding a DAP to a WAP would have a smaller effect than introducing a WAP, with an approximately 20% decrease in UMCs. We assumed that approximately 15% of the participants might fail to attend the initial telephone interview for app registration and, consequently, planned to include 280 patients in total. The initial sample size calculation was made based on separate analyses in children (n=240) and adults (n=270) [24] and further based on analysis in the whole population (n=280) owing to slow recruitment and because we expected almost similar rates of exacerbation in children and adults.

#### Analyses

Quantitative variables are described as mean (SD) or median (IQR) depending on the distribution. Between-group comparisons of continuous variables were analyzed using the nonparametric Wilcoxon 2-sample test. Qualitative variables are reported as proportions with 95% CI and compared using the chi-square test or Fisher exact test, as appropriate. As we expected some patients to experience >1 exacerbation during the follow-up and to exhibit the same behavior in terms of action plan use for each, we chose the generalized estimating equation (GEE) method to compare WAP and DAP use.

## Results

### Participants

Figure 1 presents the participant flowchart. A total of 280 participants were randomized, of whom 33 (11.8%) were excluded because of the absence of follow-up data after randomization, leaving 247 (88.2%) participants (children: n=93, 37.7%; adults: n=154, 62.3%). The WAP group included 49.8% (123/247) of participants, and the DAP+WAP group included 50.2% (124/247) of participants. Approximately three-quarters (182/247, 73.6%) of the participants were enrolled at hospitals (91 in each group), and the remaining participants were recruited by office-based physicians (WAP group: 32/123, 26%; DAP+WAP group: 33/124, 26.6%). As shown in Table 2, most patients had atopy and used maintenance treatment, and asthma control was better in children than in adults.

**Table 2:** Characteristics of the patients at inclusion (n=247).

Characteristics			WAP^a^ only (n=123)			DAP^b^+WAP (n=124)	
			Children aged 6 to 18 years (n=45)	Adults (n=78)	Children aged 6 to 18 years (n=48)		Adults (n=76)
**Sex, n (%)**							
	Female		21 (47)	56 (72)	18 (38)		59 (78)
	Male		24 (53)	22 (28)	30 (62)		17 (22)
Age (years), mean (SD)			8.3 (2.0)	36.3 (12.7)	9.0 (1.9)		34.5 (11.3)
Height (cm), mean (SD)			133.2 (11.7)	166.1 (9.9)	136.7 (12.7)		164.5 (8.3)
Weight (kg), mean (SD)			30.9 (9.5)	69.9 (19.0)	33.5 (11.2)		71.1 (18.5)
BMI (kg/m^2^), mean (SD)			17.18 (2.97)	25.32 (6.18)	17.35 (2.91)		26.25 (6.77)
Age at asthma diagnosis (years), median (IQR)			5.0 (3.0-7.0)	12.0 (5.0-24.0)	4.0 (2.0-7.0)		16.0 (4.0-30.0)
Atopy^c^, n/N (%) tested			25/38 (66)	54/72 (75)	30/42 (71)		50/69 (72)
Diagnosed with atopic dermatitis at least once, n (%)			9 (20)	23 (29)	12 (25)		21 (28)
Diagnosed with rhinitis at least once, n (%)			28 (62)	60 (77)	31 (65)		54 (71)
Diagnosed with conjunctivitis at least once, n (%)			14 (31)	34 (44)	8 (17)		34 (45)
**Smoking history, n (%)**							
	Never		—^d^	51 (65)	—		45 (56)
	Former		—	16 (21)	—		15 (20)
	Current		—	11 (14)	—		16 (21)
	Passive tobacco smoke exposure		14 (31)	—	20 (42)		—
Severe exacerbations in the past year, n			76	194	74		144
Admission for exacerbation in the past year, n (%)			21 (47)	19 (24)	21 (44)		35 (46)
Previous therapeutic education, n (%)			2 (4)	8 (10)	1 (2)		5 (7)
GINA^e^ asthma control score last month, median (IQR)			1.0 (0.0-2.0)	3.0 (1.0-4.0)	1.0 (0.0-3.0)		3.0 (1.0-4.0)
**Control, n (%)**							
	Optimal		16 (36)	16 (21)	15 (31)		15 (20)
	Partial		18 (40)	21 (27)	20 (42)		17 (22)
	Uncontrolled		11 (24)	41 (53)	13 (27)		44 (58)
**Asthma treatment, n (%)**							
	**Adherence**						
		Good	20 (44)	54 (69)	23 (48)		47 (62)
		Moderate	5 (11)	20 (26)	4 (8)		23 (30)
		Nil	0 (0)	2 (3)	1 (2)		1 (1)
		Missing^f^	20 (44)	2 (3)	20 (42)		5 (7)
	Spacer device		30 (67)	1 (1)	31 (65)		3 (4)
	Inhaled corticosteroid		19 (42)	4 (5)	17 (35)		4 (5)
	Inhaled corticosteroid plus LA^g^ β-adrenergic		19 (42)	73 (94)	22 (46)		72 (95)
	Ipratropium		0 (0)	9 (12)	0 (0)		3 (4)
	Montelukast		1 (2)	23 (29)	3 (6)		16 (21)
	Omalizumab		0 (0)	3 (4)	0 (0)		2 (3)

^a^WAP: written action plan.

^b^DAP: digital action plan.

^c^Defined as at least 1 positive skin-prick test or high antiaeroallergen immunoglobulin E titer.

^d^Not recorded.

^e^GINA: Global Initiative for Asthma.

^f^Reported by the participant or parents at the inclusion visit.

^g^LA: long-acting.

### Action Plan Use

During the 1-year follow-up, 52 (56%, 95% CI 46%-66%) of the 93 children and 89 (57.8%, 95% CI 50%-66%) of the 154 adults exhibited at least 1 exacerbation. At least 1 moderate or severe exacerbation occurred in 65 (52.8%) of the 123 WAP-group participants and 63 (50.8%) of the 124 DAP-group participants; the proportions for severe exacerbations only were 42 (34.1%) of the 123 WAP-group participants and 43 (34.7%) of the 124 DAP-group participants (*P*=.93). Overall, the mean annual rate of severe exacerbations was 0.53.

Action plan use per patient with at least 1 moderate or severe exacerbation was significantly more common in the WAP group. Results were similar when we pooled patients with mild, patients with moderate, and patients with severe exacerbations (Tables 3 and 4). Thus, the mean proportion of reported exacerbations for which the WAP was used in children and adults pooled was similar between the WAP and DAP+WAP groups (mean 39.4%, SD 44.4% vs mean 32.5%, SD 40.1%; difference −6.9%, 95% CI −21.1 to 7.2; *P*=.39 using Wilcoxon test; *P*=.95 using GEE for WAP use between the groups; and *P*=.06 for the total number of uses between the groups using GEE; the percentage of exacerbations leading to WAP use is not provided for the DAP+WAP group in Table 3). In the DAP+WAP group, the mean DAP use per reported exacerbation was 19.8% (SD 35.0%; Tables 3 and 4).

In the WAP group, the WAP was not available during 20 (12.3%) out of 163 exacerbations (limitation for delivery at scale), and the main reason for not using the WAP was perceived uselessness given the severity of the exacerbation. In the DAP+WAP group, the WAP was not available for 13 (8.6%) out of 151 exacerbations, and either the smartphone or an internet connection was not available for 12 (7.9%) out of 151 exacerbations.

**Table 3:** Outcomes in the overall population during the 1-year follow-up (n=247).

Outcomes					WAP^a^ only (n=123)						DAP^b^+WAP (n=124)							*P* value
					Children aged 6 to 12 years (n=45)			Adults (n=78)			Children aged 6 to 12 years (n=48)			Adults (n=76)			WAP versus DAP+WAP	
Patients with at least 1 reported exacerbation^c^, n (%)					25 (56)			48 (62)			27 (56)			71 (54)			.47^d^	
**Exacerbations, n^e^**					53			110			56			95			—^f^	
	Patients with 0 exacerbation, n (%)			20 (44)			30 (38)			21 (44)			35 (46)					
	Patients with 1 exacerbation, n (%)			13 (29)			21 (27)			12 (25)			14 (18)					
	Patients with 2 exacerbations, n (%)			4 (9)			13 (17)			8 (17)			14 (18)					
	Patients with 3 exacerbations, n (%)			4 (9)			6 (8)			3 (6)			6 (8)					
	Patients with >3 exacerbations, n (%)			4 (9)			8 (10)			4 (8)			7 (9)					
Number of exacerbations per patient, median (IQR)					1.0 (0.0-2.0)			1.0 (0.0-2.0)			1.0 (0.0-2.0)			1.0 (0.0-2.0)			.68^g^	
**Unscheduled medical contacts, n^e^**					12			26			13			33			.82^g^	
	Patients with 0 unscheduled medical contacts, n (%)			34 (76)			58 (74)			40 (83)			56 (74)					
	Patients with 1 unscheduled medical contact, n (%)			10 (22)			16 (21)			5 (10)			12 (16)					
	Patients with 2 unscheduled medical contacts, n (%)			1 (2)			3 (4)			2 (4)			4 (5)					
	Patients with 3 unscheduled medical contacts, n (%)			0 (0)			0 (0)			0 (0)			3 (4)					
	Patients with 4 unscheduled medical contacts, n (%)			0 (0)			1 (1)			1 (2)			1 (1)					
Unscheduled medical contacts, n of patients (%)					11 (24)			20 (26)			8 (17)			20 (26)			.63^d^	
**Level of asthma control, n (%)**																		
	**3 months**																.049^d^	
		Optimal	36 (80)			42 (54)			34 (71)			34 (45)						
		Partial	3 (7)			17 (22)			8 (17)			26 (34)						
		Uncontrolled	5 (11)			17 (22)			2 (4)			12 (16)						
	**6 months**																.87^d^	
		Optimal	36 (80)			43 (55)			34 (71)			43 (57)						
		Partial	3 (7)			14 (18)			5 (10)			9 (12)						
		Uncontrolled	4 (9)			16 (21)			3 (6)			14 (18)						
	**9 months**																.51^d^	
		Optimal	36 (80)			45 (58)			33 (69)			43 (57)						
		Partial	5 (11)			11 (14)			6 (12)			13 (17)						
		Uncontrolled	3 (7)			15 (19)			2 (4)			10 (13)						
	**12 months**																.40^d^	
		Optimal	33 (73)			34 (44)			29 (60)			36 (47)						
		Partial	2 (4)			18 (23)			6 (12)			19 (25)						
		Uncontrolled	6 (13)			16 (21)			5 (10)			10 (13)						
Exacerbations recorded in the DAP, n^e^ (patients)					—			—			9 (8)			24 (17)			—	
**Action plan use during the 1-year follow-up^h^**																		
	Action plan uses/reported exacerbations, n/N (%)			29/53 (55)			35/110 (32)			9/55 (16)			24/92 (26)			.03^i^		
	**Intergroup comparison according to the use of action plan**																.02^g^	
		Patients who never used the action plan, n (%)	9 (20)			29 (37)			19 (40)			29 (38)						
		Patients who used the action plan once, n (%)	8 (18)			11 (14)			5 (10)			7 (9)						
		Patients who used the action plan twice, n (%)	4 (9)			3 (4)			2 (4)			2 (3)						
		Patients who used the action plan 3 times, n (%)	3 (7)			3 (4)			0 (0)			1 (1)						
		Patients who used the action plan 4 times, n (%)	1 (2)			1 (1)			0 (0)			1 (1)						
		Patients who used the action plan 5 times, n (%)	0 (0)			1 (1)			0 (0)			0 (0)						
		Patients who used the action plan 6 times, n (%)	0 (0)			0 (0)			0 (0)			1 (1)						
		Action plan uses in patients with exacerbations, n/N (%)	16/25 (64)			19/48 (40)			7/26 (27)			12/41 (29)			.02^d^			
**Percentage of exacerbations leading to action plan use, n (%)**																		.03^j^
	0			9 (36)			29 (60)			19 (73)			29 (71)					
	0-50			2 (8)			6 (13)			3 (12)			6 (15)					
	50-100			3 (12)			3 (6)			1 (4)			1 (2)					
	100			11 (44)			10 (21)			3 (12)			5 (12)					
**Subjective evaluation of action plan^h^**																		
	Patients who evaluated, n (%)			16 (36)			22 (28)			8 (17)			17 (22)			—		
	**Score on 1-10** **VAS^k,l^, median (IQR)**																	
		Availability	10.0 (9.5-10.0)			10.0 (8.0-10.0)			10.0 (6.0-10.0)			10.0 (8.0-10.0)			.39^g^			
		Usefulness	10.0 (8.5-10.0)			10.0 (10.0-10.0)			10.0 (8.0-10.0)			9.0 (7.0-10.0)			.26^g^			
		Avoided unscheduled medical contact	9.0 (7.5-10.0)			10.0 (7.0-10.0)			10.0 (5.0-10.0)			9.5 (2.5-10.0)			.62^g^			

^a^WAP: written action plan.

^b^DAP: digital action plan.

^c^Mild, moderate, or severe exacerbation.

^d^Chi-square test.

^e^Raw value without available denominator (no percentage).

^f^Not available.

^g^Wilcoxon test.

^h^Only DAP was considered in the DAP+WAP group.

^i^Generalized estimating equation.

^j^Fisher exact test.

^k^VAS: visual analog scale.

^l^The participants used the visual analog scale at last follow-up 1 year after study inclusion.

**Table 4:** Outcomes in the 128 participants with at least 1 moderate or severe exacerbation during the 1-year follow-up.

Outcomes			WAP^a^ only (n=65)			DAP^b^+WAP (n=63)			*P* value
			Children aged 6 to 12 years (n=21)	Adults (n=44)	Children aged 6 to 12 years (n=24)		Adults (n=39)	WAP versus DAP+WAP	
**Exacerbations, n^c^**			44	92	45		74	—^d^	
	Patients with 1 exacerbation, n (%)		9 (43)	21 (48)	11 (46)		17 (47)		
	Patients with 2 exacerbation, n (%)		5 (24)	13 (30)	8 (33)		15 (42)		
	Patients with 3 exacerbations, n (%)		5 (24)	5 (11)	2 (8)		2 (6)		
	Patients with >3 exacerbations, n (%)		2 (10)	5 (11)	3 (8)		5 (14)		
Number of exacerbations per patient, median (IQR)			2.0 (1.0-3.0)	2.0 (1.0-2.0)	2.0 (1.0-2.0)		2.0 (1.0-2.0)	.81^e^	
**Unscheduled medical contacts, n^c^**			12	25	12		33	.85^e^	
	Patients with 0 unscheduled medical contact, n (%)		10 (48)	25 (57)	17 (71)		19 (49)		
	Patients with 1 unscheduled medical contact, n (%)		10 (48)	15 (34)	4 (17)		12 (31)		
	Patients with 2 unscheduled medical contacts, n (%)		1 (5)	3 (7)	2 (8)		4 (10)		
	Patients with 3 unscheduled medical contacts, n (%)		0 (0)	0 (0)	0 (0)		3 (8)		
	Patients with 4 unscheduled medical contacts, n (%)		0 (0)	1 (2)	1 (4)		1 (3)		
Unscheduled medical contacts, n of patients (%)			11 (52)	19 (43)	7 (29)		20 (51)	.71^f^	
**Level of asthma control, n (%)**									
	**3 months**								.60^f^
		Optimal	15 (71)	17 (39)	17 (71)		13 (33)		
		Partial	2 (10)	12 (27)	4 (17)		13 (33)		
		Uncontrolled	4 (19)	14 (32)	2 (8)		11 (14)		
	**6 months**								.83^f^
		Optimal	17 (81)	21 (48)	16 (67)		17 (44)		
		Partial	0 (0)	7 (16)	2 (8)		6 (15)		
		Uncontrolled	3 (14)	15 (34)	3 (12)		11 (28)		
	**9 months**								.62^f^
		Optimal	14 (67)	22 (50)	17 (71)		19 (49)		
		Partial	3 (14)	8 (18)	2 (8)		5 (13)		
		Uncontrolled	3 (14)	12 (27)	2 (8)		9 (23)		
	**12 months**								.32^f^
		Optimal	14 (67)	19 (43)	13 (54)		16 (41)		
		Partial	1 (5)	9 (20)	5 (21)		10 (26)		
		Uncontrolled	5 (24)	13 (30)	5 (21)		7 (18)		
**Exacerbations recorded in the DAP, n^c^ (patients)**			—	—	14 (10)		25 (17)	—	
	Moderate exacerbations		—	—	5		15		
	Severe exacerbations		—	—	4		9		
**Action plan use during the 1-year follow-up^g^**									
	Action plan uses/reported exacerbations, n/N (%)		27/44 (61)	28/92 (30)	5/45 (11)		14/74 (19)	.001^h^	
	**Intergroup comparison according to the use of action plan**								.001^e^
		Patients who never used the action plan, n (%)	6 (29)	26 (59)	19 (79)		29 (74)		
		Patients who used the action plan once, n (%)	7 (33)	12 (27)	5 (21)		7 (18)		
		Patients who used the action plan twice, n (%)	4 (19)	4 (9)	0 (0)		2 (5)		
		Patients who used the action plan 3 times, n (%)	4 (19)	1 (2)	0 (0)		1 (3)		
		Patients who used the action plan 4 times, n (%)	0 (0)	0 (0)	0 (0)		0 (0)		
		Patients who used the action plan 5 times, n (%)	0 (0)	1 (2)	0 (0)		0 (0)		
		Action plan uses in patients with exacerbations, n/N (%)	15/21 (71)	18/44 (41)	5/24 (21)		10/39 (26)	.002^f^	
**Percentage of exacerbations leading to action plan use, n (%)**									.001^i^
	0		6 (29)	26 (59)	19 (79)		29 (74)		
	0-50		1 (5)	5 (11)	3 (13)		4 (10)		
	50-100		2 (10)	3 (7)	0 (0)		0 (0)		
	100		12 (57)	10 (23)	2 (8)		6 (15)		
**Subjective evaluation of action plan**									
	Patients who evaluated, n (%)		15 (71)	7 (16)	6 (25)		12 (31)	—	
	**Score on 1-10** **VAS^j^** **, median (IQR)**								
		Availability	10.0 (10.0-10.0)	10.0 (8.0-10.0)	8.5 (5.0-10.0)		10.0 (8.5-10.0)	.52^e^	
		Usefulness	10.0 (9.0-10.0)	10.0 (10.0-10.0)	10.0 (6.0-10.0)		9.0 (7.5-10.0)	.19^e^	
		Avoided unscheduled medical contact	9.0 (7.0-10.0)	10.0 (6.0-10.0)	9.0 (5.0-10.0)		9.5 (0.0-10.0)	.56^e^	

^a^WAP: written action plan.

^b^DAP: digital action plan.

^c^Raw value without available denominator (no percentage).

^e^Descriptive data.

^f^Wilcoxon test.

^g^Chi-square test.

^h^Only DAP was considered in the DAP+WAP group.

^i^Generalized estimating equation.

^j^Fisher exact test.

^k^VAS: visual analog scale.

### Web App Connections

Of the 38 DAP connections, 5 (13%) failed to establish severity owing to inconsistencies (limitation for delivery at scale). These 5 connections were identified when the participants or parents reported DAP use, but no connection was recorded in the app. Furthermore, 33 connections to the app for 27 exacerbations were recorded, 9 by parents for 8 exacerbations and 24 by adults for 17 exacerbations. The median number of descriptors entered into the app was 5 [3-8].

At connection, the app classified the exacerbation as mild in 12 cases, moderate in 11 cases, and severe in 10 cases. Two examples of advice provided by the app are given in Multimedia Appendix 2.

**Figure 2:**
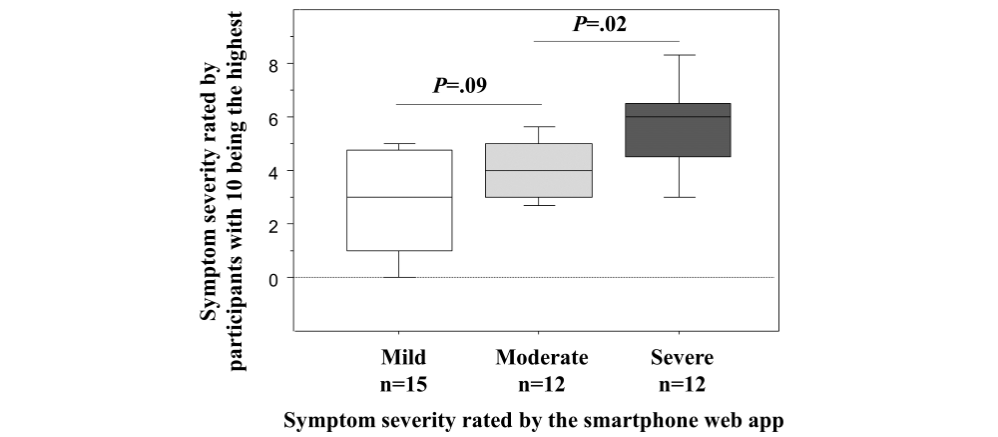
Symptom severity as evaluated by the participants and by the app. Before recording the symptoms of asthma, the participant or parent recorded the symptom severity in the app (see the Methods section: from 0=no exacerbation to 10=maximal severity). Once the symptoms were recorded, the app categorized their severity as mild, moderate, or severe (x-axis). Severity as assessed by the participants or parents was consistent with the severity as assessed by the app (3-group comparison using the Kruskal-Wallis test: *P*=.001). The *P* values in the figure are those obtained using the Mann-Whitney U test for 2-group comparisons.

Median symptom severity self-evaluated by the participants on a 10-point scale before recording the symptoms in the app was 4 [3-6] and was not significantly different from the symptom severity assessed by the app (Figure 2). The number of connections recorded in the app was consistent with the number subsequently reported during the telephone interviews. For 5 (13%) of the 38 connections, the DAP failed to categorize exacerbation severity owing to incoherent symptoms. The main reason for not using the DAP was mildness of the symptoms, which made assistance for management unnecessary.

### Number of UMCs

The number of UMCs was 25.2% (31/123) in the WAP group and 22.6% (28/123) in the DAP+WAP group (difference −2.6%, 95% CI −13.3% to 8.0%; *P*=.63). The mean number of UMCs during the year in these 2 groups was 0.30 (SD 0.62) and 0.37 (SD 0.82), respectively (mean difference 0.06, 95% CI −0.12 to 0.24; *P*=.82). The number of UMCs was not significantly different between the 2 groups, regardless of whether we considered all exacerbations (mild to severe; Table 3) or only moderate and severe exacerbations (Table 4).

Overall, the findings were similar between children and adults. Participant satisfaction with the action plans was very high and not significantly different between the WAP and DAP+WAP groups.

## Discussion

### Principal Findings

The main finding from this multicenter randomized controlled trial is that a DAP combined with a WAP for the self-management of asthma exacerbations did not decrease the number of UMCs compared with the WAP alone. Participant satisfaction was similar with the 2 interventions. Most participants used no action plan when exacerbations occurred, and the DAP was used less often than the WAP.

### Comparison With Prior Work

Our participants had typical asthma, usually with an allergic component, and the vast majority was on asthma controller therapy. As expected, the exacerbation rate was similar between the 2 groups during follow-up. Nevertheless, only approximately half (141/247, 57.1%) of the participants experienced exacerbations, whereas we expected a proportion of 80% based on the literature [22,23]. This lower proportion may be ascribed to good asthma management throughout the follow-up. The frequency of severe exacerbations in our study is consistent with that in 2 recent multicenter trials [25,26] and is well above the rate between 0.1 and 0.2 per patient per year in unselected populations of patients with asthma [27]. These rates of exacerbations were similar between our 2 groups of participants; thus, DAP provision did not nudge the patients to seek help.

Systematic reviews of smartphone self-management apps and tablet self-management applications for asthma available in 2013 and 2019, respectively, suggested that the current evidence was insufficient to make clinical recommendations [12,28]. We initially hypothesized that the DAP might decrease UMCs for 2 reasons. First, patients might leave their WAP at home when they go out but take their smartphone with them, making the DAP more often available should symptoms occur. Second, we also believed that the ability to connect several times during the same exacerbation and, thus, to obtain instructions consistent with the changing clinical picture might be both more effective and more reassuring than the WAP, as suggested by a recent study [18]. The ability to reassure and provide advice over time was important, as parental anxiety or fear was the most common reason for avoidable UMCs in an earlier pediatric study [7]. Nonetheless, UMCs were not less common in the DAP+WAP group compared with the WAP group. WAP use was similar between the 2 groups, and the DAP was used less often than the WAP. Overall, our results are truly negative, demonstrating no favorable trend for DAP versus WAP use even if digital health interventions show substantial promise for asthma disease monitoring and the personalization of treatment [29].

Our study extends the results of previous trials devoted to improving asthma control [10,11], showing that even when the need for urgent advice is felt, the use of a DAP was restricted, at least in a French health care system in which UMCs are easily obtained.

Many parents and patients did not use the action plans. It has been stated that professionally provided medically focused action plans that do not fit with and incorporate the patient’s and carer’s views of asthma and their management strategies will continue to be underutilized [30]. By contrast, the patients could have been insufficiently educated to understand the potential benefits of action plans. This underuse may also reflect the patients’ confidence in their ability to manage their exacerbations without advice. Their evaluation of exacerbation severity done when using the app was reasonably accurate. A 2020 French survey found that only 54% of parents would use an algorithmic decision system for managing their child’s asthma [31]. Similarly, a study using semistructured interviews showed that patients were not confident that artificial intelligence could generate new advice or reach diagnostic conclusions without the interpretation of their trusted clinicians [17]. Moreover, whether a WAP produces added benefits in patients who receive asthma education is debated [3]. All the physicians in our study were asthma specialists who delivered asthma education. In patients with asthma seen for the first time by specialists, a WAP did not decrease UMCs compared with no WAP [6]. Both groups showed similar significant reductions in asthma symptoms, as observed in our study. Thus, asthma education in any form may be an effective intervention. Even when a WAP is given, UMCs for asthma exacerbations are associated with several factors, including the number of UMCs in the past year [7].

### Limitations

The first limitation of our study was that it was not certain whether all participants were asthmatic, as the evidence of variable expiratory airflow limitation was not an inclusion criterion. Nevertheless, inclusion and noninclusion criteria allowed being confident with the inclusion of participants who were truly asthmatic, mainly those who were atopic; moreover, the severe exacerbation rate observed was in accordance with previous studies. The second limitation of our trial was the provision of the WAP to all patients. Not providing a WAP would have been incompatible with the current guidelines, and we could not be sure that an internet connection would always be available. Third, WAP use was self-reported every 3 months and may, therefore, have been subject to recall bias. As for the DAP, the number of connections recorded in the app was consistent with the number reported during the telephone interviews. Fourth, all patients received the same WAP, which complied with French recommendations, but developing WAPs in discussion with each patient might improve effectiveness. However, our study assessed whether adding a DAP was beneficial, and the characteristics of the WAP could not have affected our findings. Fifth, neither the education session delivered at the time the WAP was provided nor the follow-up by physicians was standardized, which could have modified the asthma education received by patients and families. Sixth, the numbers of children and adults were not sufficient for performing separate statistical analyses in the 2 age groups; such analyses were planned initially [24] but not performed owing to slow recruitment. Seventh, a limitation to recruitment was that we included only patients who had no WAP. As WAP delivery was widespread at least in France [7,32] in keeping with guidelines, this criterion decreased the pool of eligible patients. Eighth, our results are generalizable only to patients with asthma who benefited from an action plan provision and related explanations, which is important to highlight because the use of action plan is somewhat restricted: 30.6% of allergists and pulmonologists in a US survey [33]. Ninth, we could not include patients aged 13 to 17 years, as regulations prohibited the independent use of the DAP in this age group.

### Conclusions

The DAP was used less often than the WAP by both adults and parents of children with asthma, and having a DAP did not decrease the number of UMCs in the specific context of our study before the new guidelines recommending the addition or the increase in inhaled corticosteroid treatment, which does not call into question the interest of the action plan and its associated therapeutic education. The benefits of tele–health care in asthma care remain to be demonstrated.

## Appendix

Multimedia Appendix 1 Recording of the participant, first connection, and recording of an asthma attack.

Multimedia Appendix 2 Two examples of advice provided by the app.

Multimedia Appendix 3 CONSORT-EHEALTH (V 1.6.1).

## Abbreviations

DAP: digital action plan
GEE: generalized estimating equation
GINA: Global Initiative for Asthma
UMC: unscheduled medical contact
VAS: visual analog scale
WAP: written action plan

## Data Availability

The data sets generated or analyzed during this study are available from the corresponding author upon reasonable request. Mosquitom has protected the algorithm used in the web app.
