# Supplementary material for: Digital Action Plan (Web App) for Managing Asthma Exacerbations: Randomized Controlled Trial
Source: J Med Internet Res. 2023 Jun 29;25:e41490. doi: 10.2196/41490 (PMC10365576; doi:10.2196/41490)
Supplement: Multimedia Appendix 1 [file jmir_v25i1e41490_app1.pdf]

Recording of a participant by the research nurse

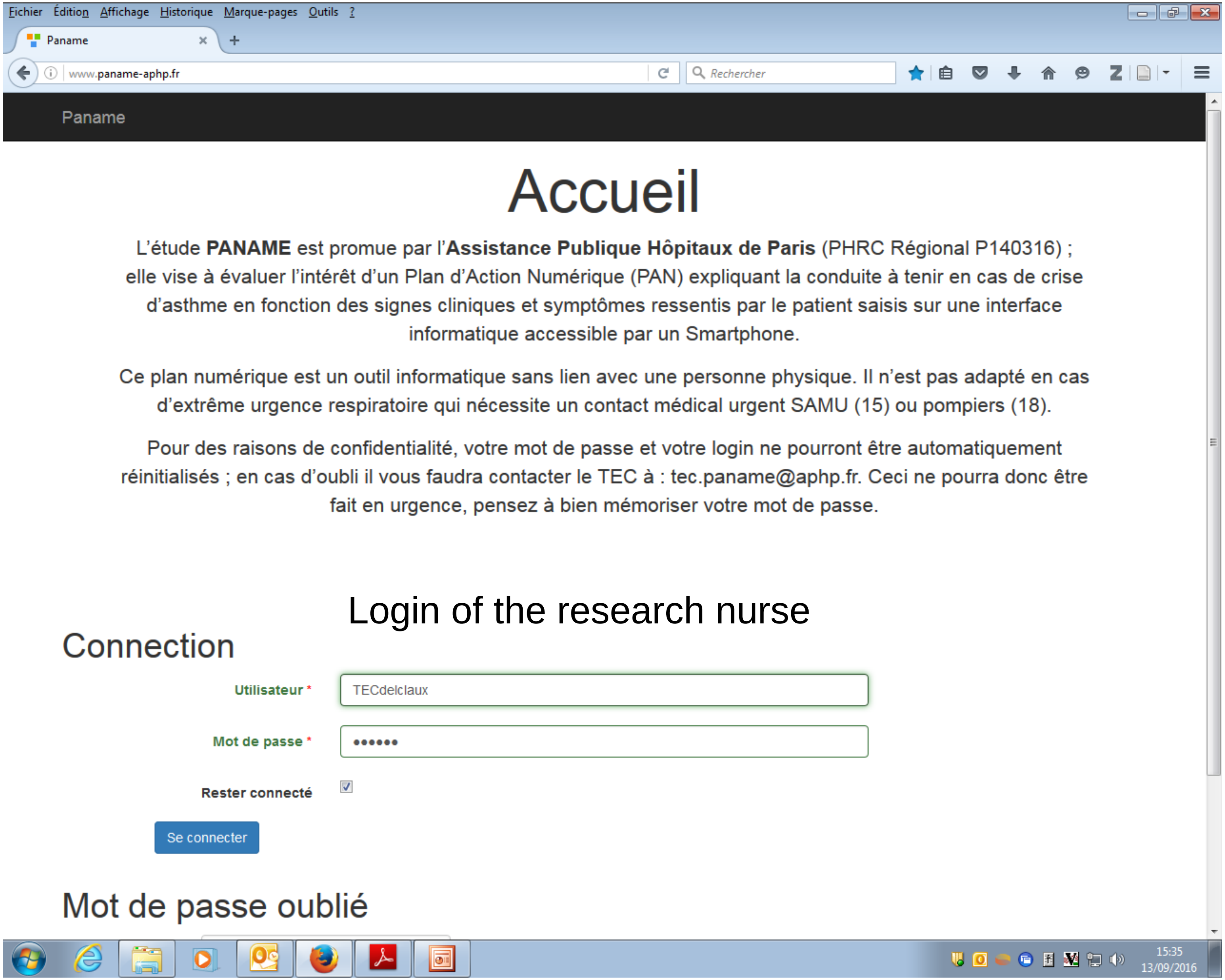

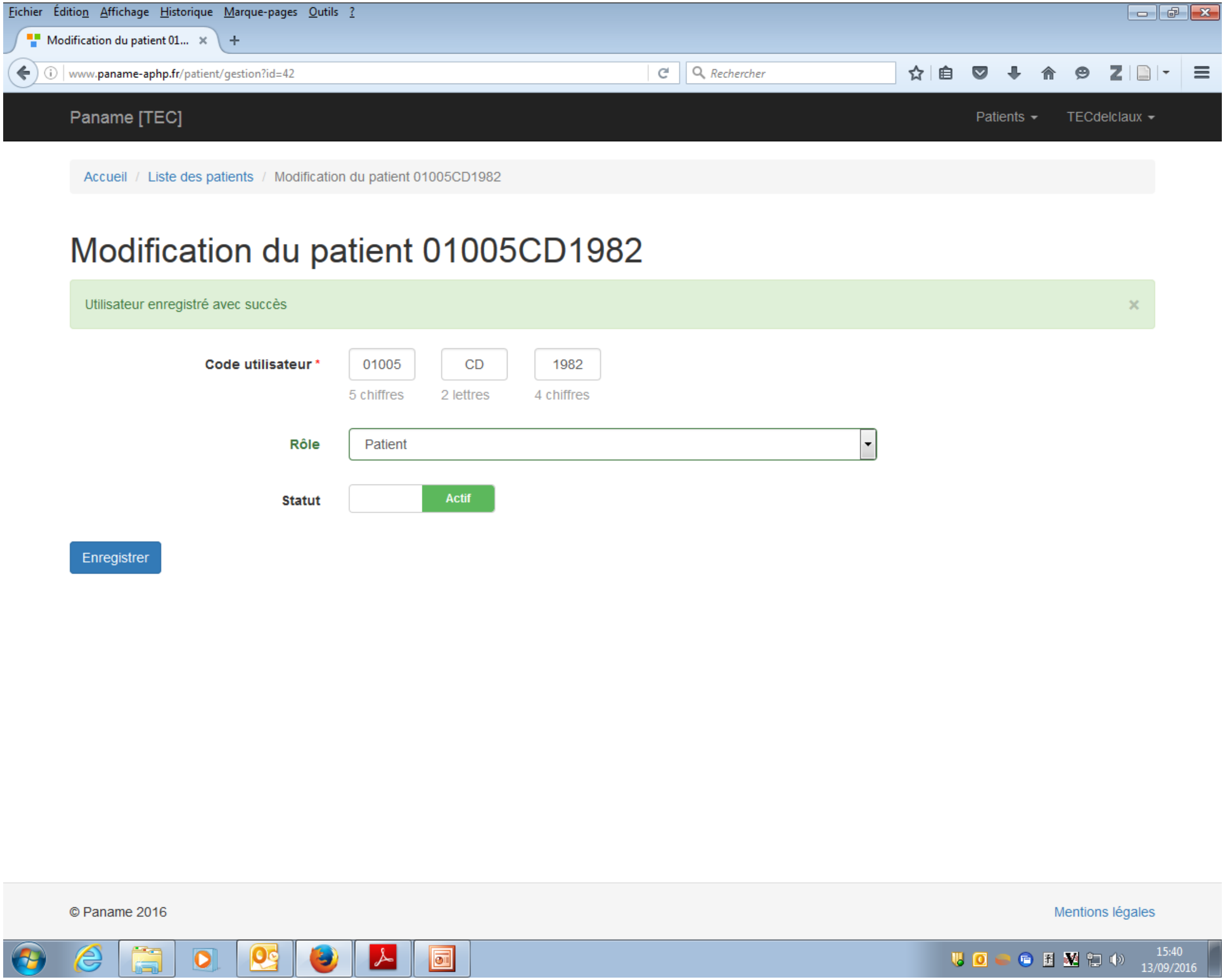
Fichier Édition Affichage Historique Marque-pages Outils ?
Modification du patient 01...
www.paname-aphp.fr/patient/gestion?id=42
Rechercher
Paname [TEC]
Patients
TECdelclaux
Accueil / Liste des patients / Modification du patient 01005CD1982
Modification du patient 01005CD1982
Utilisateur enregistré avec succès
Code utilisateur *
01005
CD
1982
5 chiffres
2 lettres
4 chiffres
Rôle
Patient
Statut
Actif
Enregistrer
© Paname 2016
Mentions légales
15:40
13/09/2016

Fichier Édition Affichage Historique Marque-pages Outils ?

Dossier médical de 01005C...

www.paname-aphp.fr/utilisateurs/dossier?id=42

Rechercher

Paname [TEC]   Patients   TECdelclaux

Accueil / Liste des patients / Dossier médical de 01005CD1982

Dossier médical de 01005CD1982

	Médicament	Posologie
**Bronchodilatateur [[BD]]**	Ventoline	
**Corticoïde oral [[CO]]**	Solupred	60 mg (3 comprimés à 20 mg)
**Chambre d'inhalation [[CH]]**		
**Glucocorticoïde [[GC]]**	Innovair	1 prise matin et soir
**Plan d'action numérique**	Adulte	

Enregistrer

The nurse has to postpone the drugs
(names of drugs understandable to patient)
that appear on the inclusion sheet
then "save"

© Paname 2016

Mentions légales

15:56
13/09/2016

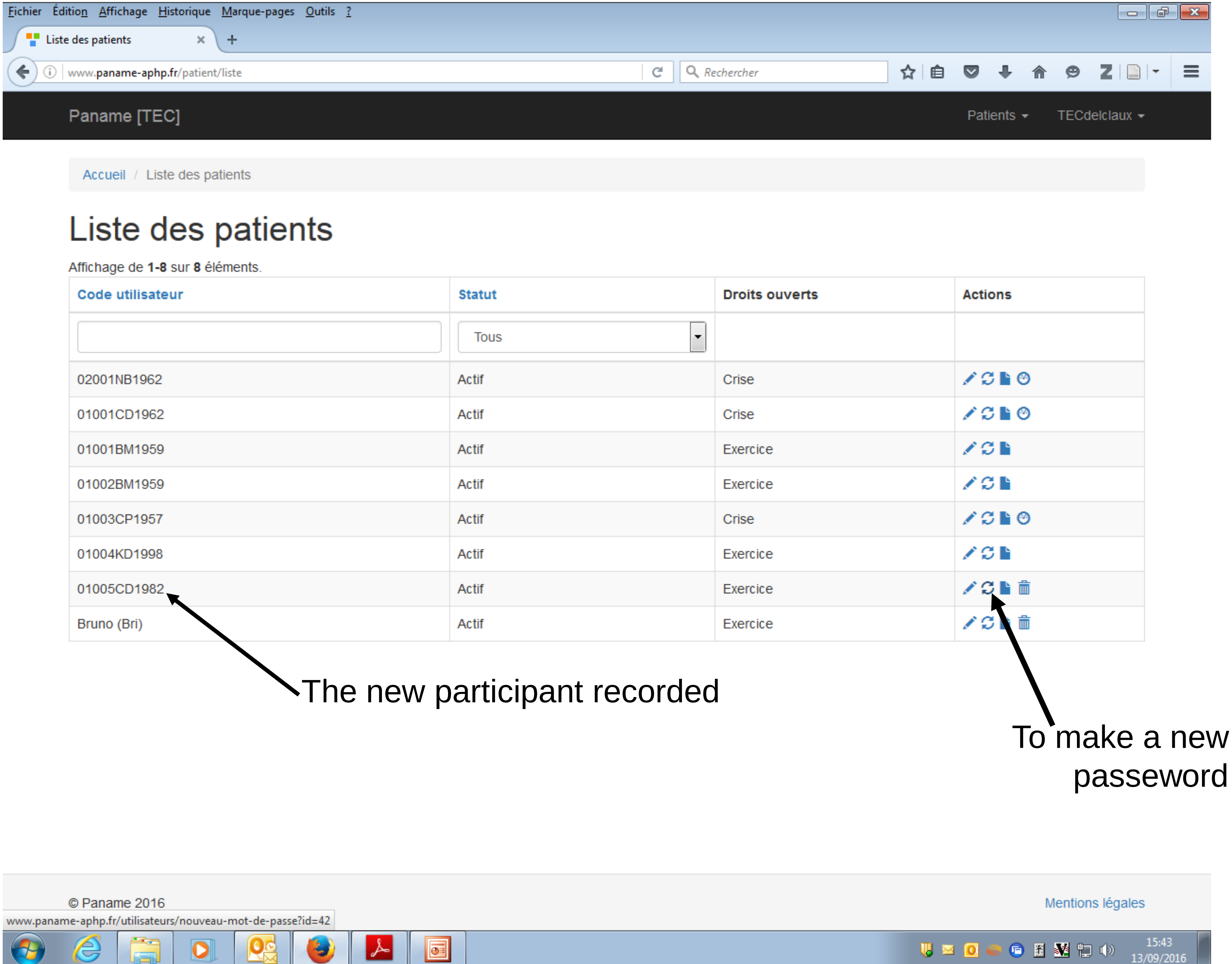
Fichier Édition Affichage Historique Marque-pages Outils ?
Liste des patients
www.paname-aphp.fr/patient/liste
Rechercher
Paname [TEC]
Patients
TECdelclaux
Accueil / Liste des patients
Liste des patients
Affichage de 1-8 sur 8 éléments.
Code utilisateur
Statut
Droits ouverts
Actions
Tous
02001NB1962 Actif Crise
01001CD1962 Actif Crise
01001BM1959 Actif Exercice
01002BM1959 Actif Exercice
01003CP1957 Actif Crise
01004KD1998 Actif Exercice
01005CD1982 Actif Exercice
Bruno (Bri) Actif Exercice
The new participant recorded
To make a new passeword
© Paname 2016
Mentions légales
www.paname-aphp.fr/utilisateurs/nouveau-mot-de-passe?id=42
15:43
13/09/2016

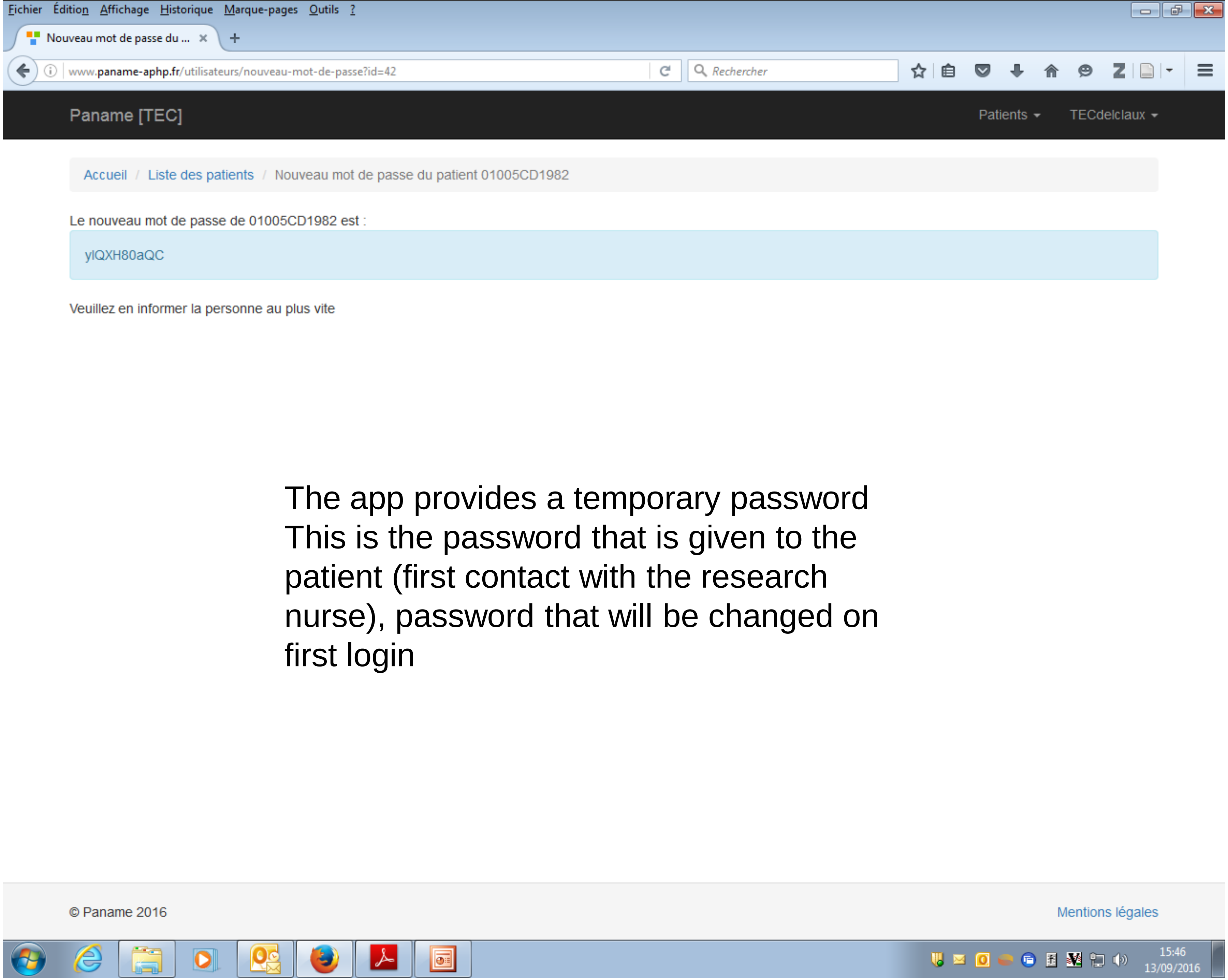

The app provides a temporary password
This is the password that is given to the patient (first contact with the research nurse), password that will be changed on first login

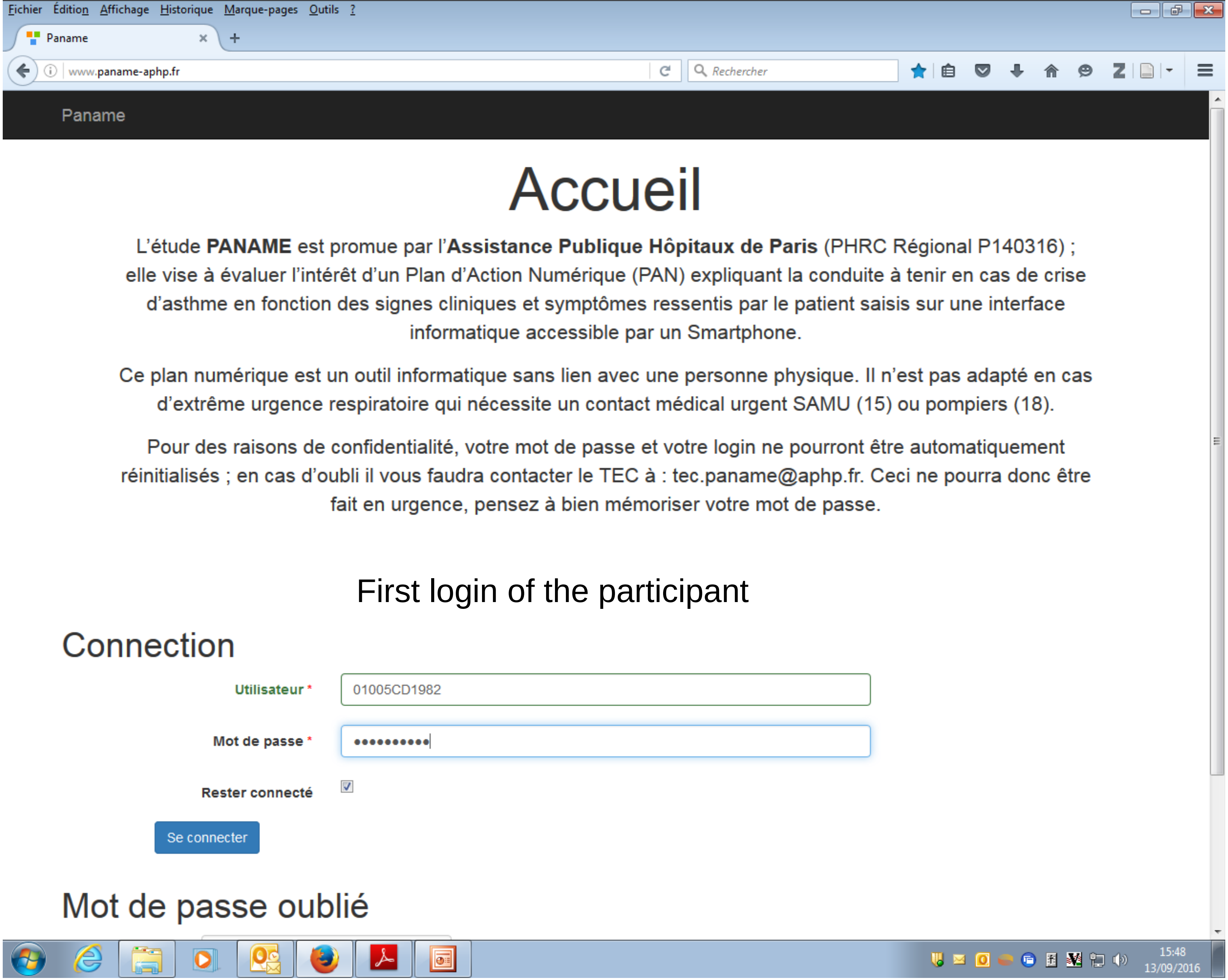
Fichier Édition Affichage Historique Marque-pages Outils ?
Paname
www.paname-aphp.fr
Rechercher
Paname
Accueil
L'étude PANAME est promue par l'Assistance Publique Hôpitaux de Paris (PHRC Régional P140316) ; elle vise à évaluer l'intérêt d'un Plan d'Action Numérique (PAN) expliquant la conduite à tenir en cas de crise d'asthme en fonction des signes cliniques et symptômes ressentis par le patient saisis sur une interface informatique accessible par un Smartphone.
Ce plan numérique est un outil informatique sans lien avec une personne physique. Il n'est pas adapté en cas d'extrême urgence respiratoire qui nécessite un contact médical urgent SAMU (15) ou pompiers (18).
Pour des raisons de confidentialité, votre mot de passe et votre login ne pourront être automatiquement réinitialisés ; en cas d'oubli il vous faudra contacter le TEC à : tec.paname@aphp.fr. Ceci ne pourra donc être fait en urgence, pensez à bien mémoriser votre mot de passe.
First login of the participant
Connection
Utilisateur *
01005CD1982
Mot de passe *
Rester connecté
Se connecter
Mot de passe oublié
15:48
13/09/2016

Paname [Patient]

Accueil / Changement de mot de passe

Changement de mot de passe

Nouveau mot de passe *

Confirmer le nouveau mot de passe *

Enregistrer

The patient enters a new password
Easiest to remember (no password security requirements)
He/she can also be told to have his login (username) and password memorized by his/her phone

© Paname 2016

Mentions légales

Fichier Édition Affichage Historique Marque-pages Outils ?

Paname

www.paname-aphp.fr

Rechercher

Paname [Patient] Exercice 01005CD1982

Bonjour 01005CD1982

Je veux m'exercer
avec le plan d'action numérique

First connection

An exercise has to be done, training session (validation process)

© Paname 2016

Mentions légales

16:00
13/09/2016

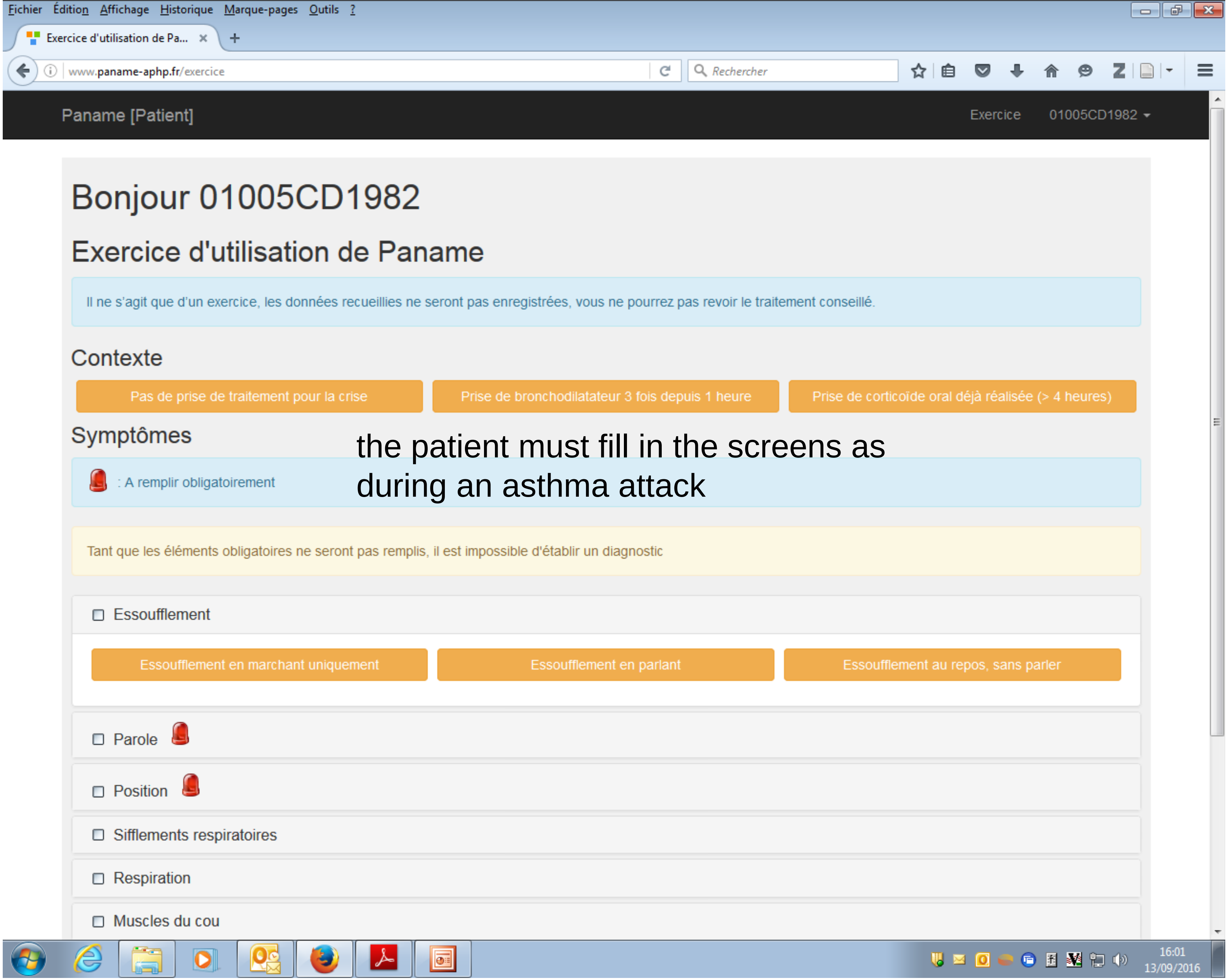

the patient must fill in the screens as during an asthma attack

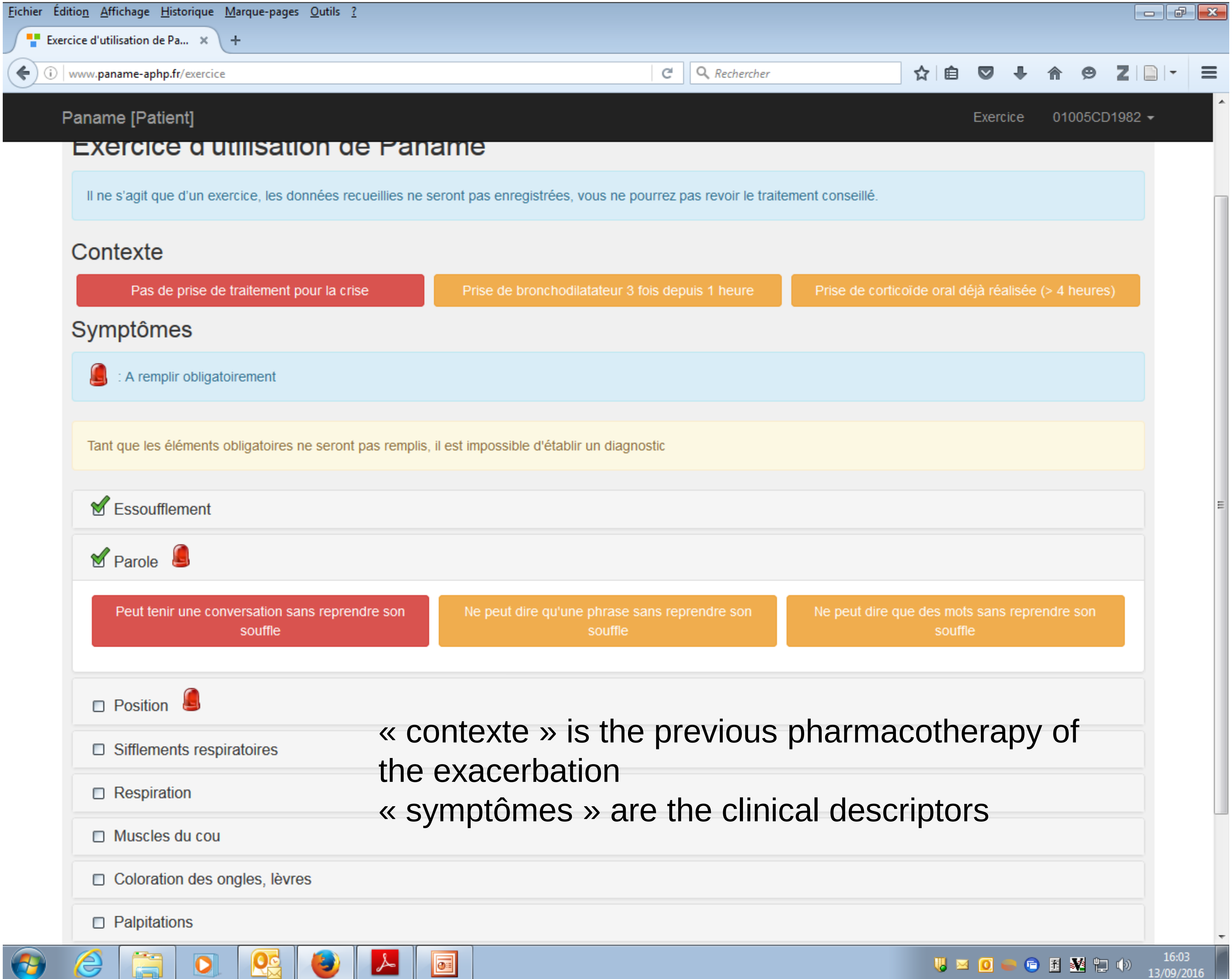

« contexte » is the previous pharmacotherapy of the exacerbation
« symptômes » are the clinical descriptors

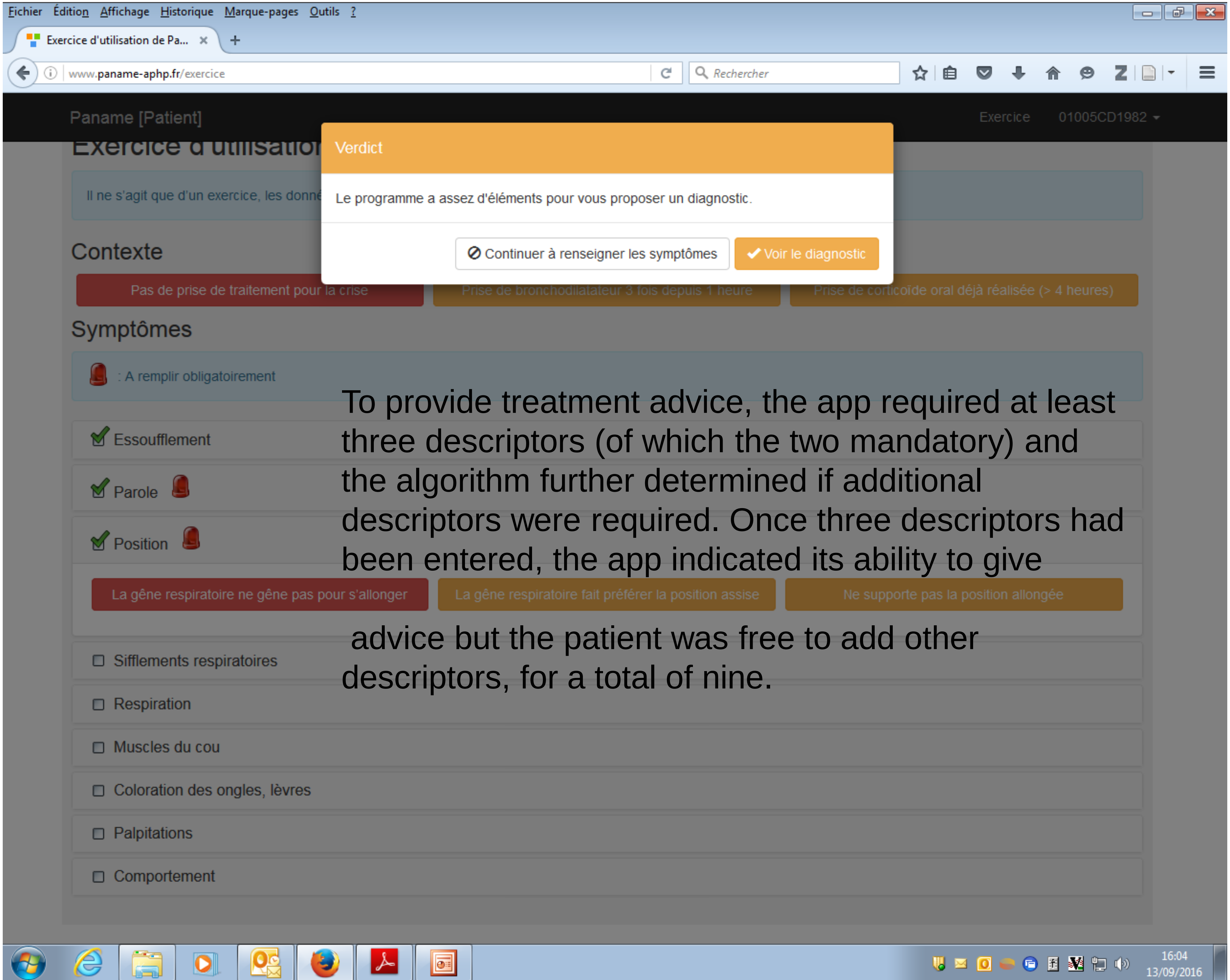

To provide treatment advice, the app required at least three descriptors (of which the two mandatory) and the algorithm further determined if additional descriptors were required. Once three descriptors had been entered, the app indicated its ability to give advice but the patient was free to add other descriptors, for a total of nine.

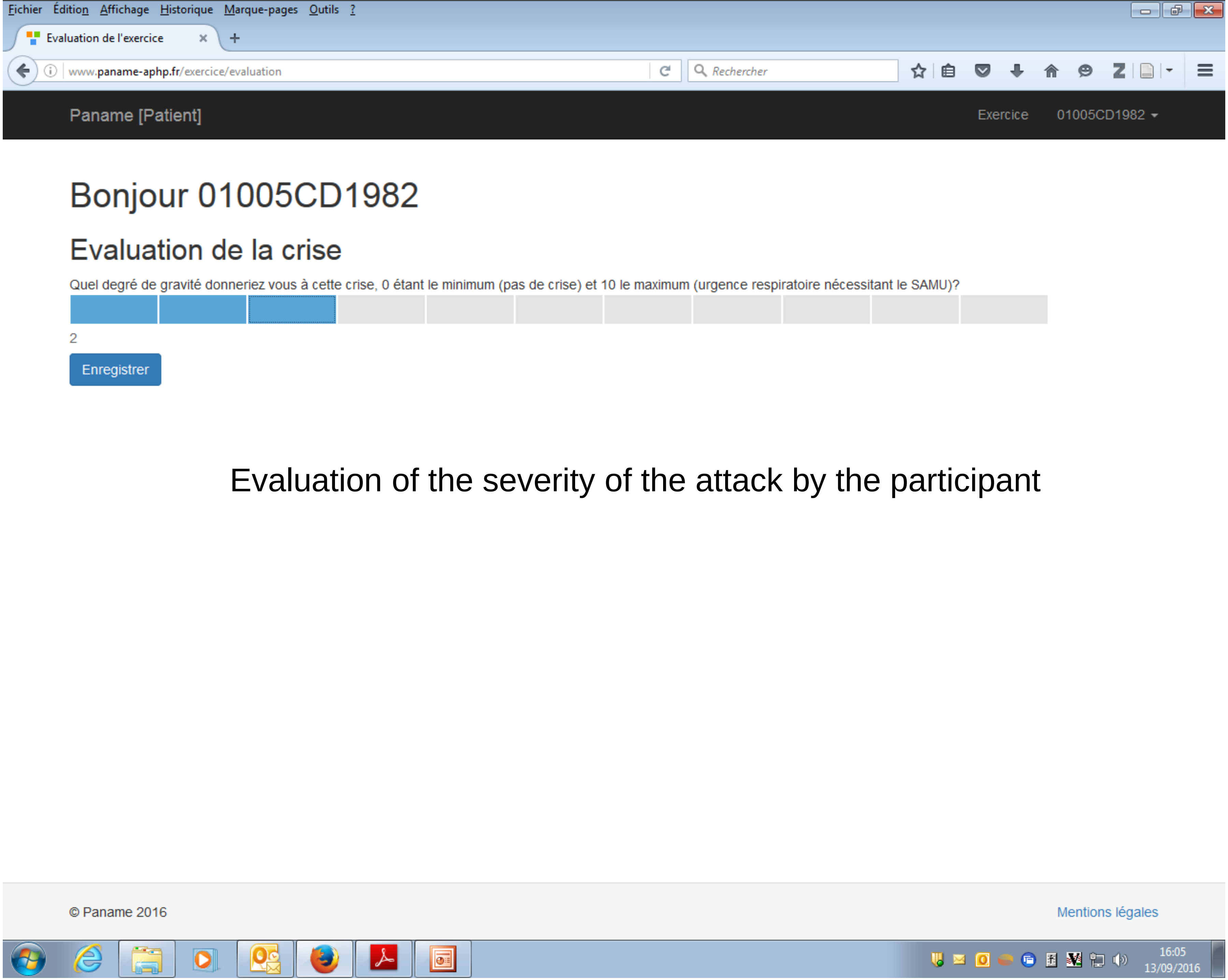

Evaluation of the severity of the attack by the participant

Fichier Édition Affichage Historique Marque-pages Outils ?

Diagnostic de crise

www.paname-aphp.fr/exercice/diagnostic

Rechercher

Paname [Patient]

Crise Historique des crises Exercice 01005CD1982

Bonjour 01005CD1982

Diagnostic de crise

Vous avez effectué un exercice.
Vous avez maintenant accès à la déclaration de crise.

the training session is completed
No diagnosis or treatment is given but now
the application is functional
The nurse receives an email

© Paname 2016

Mentions légales

16:06
13/09/2016

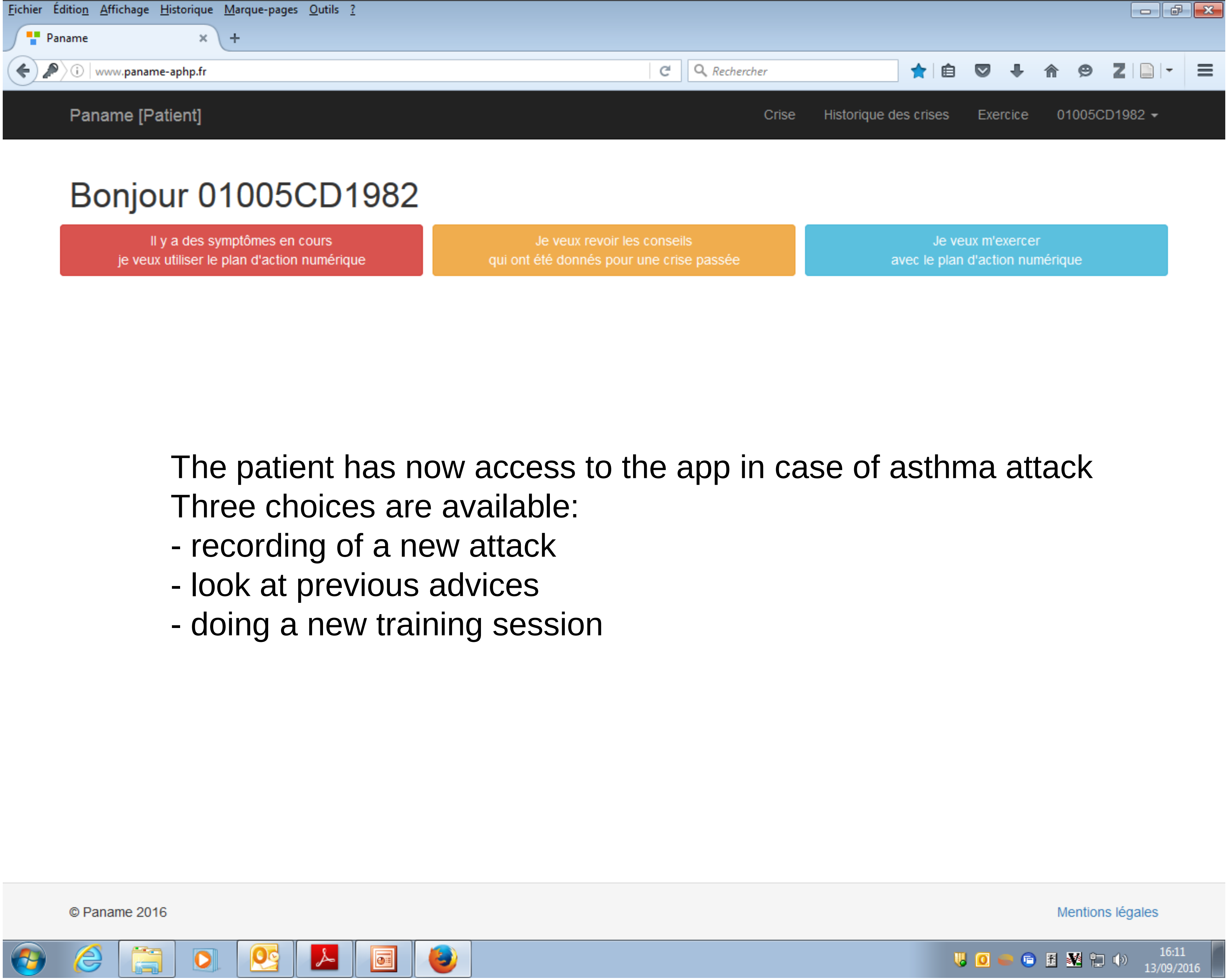

The patient has now access to the app in case of asthma attack
Three choices are available:
- recording of a new attack
- look at previous advices
- doing a new training session

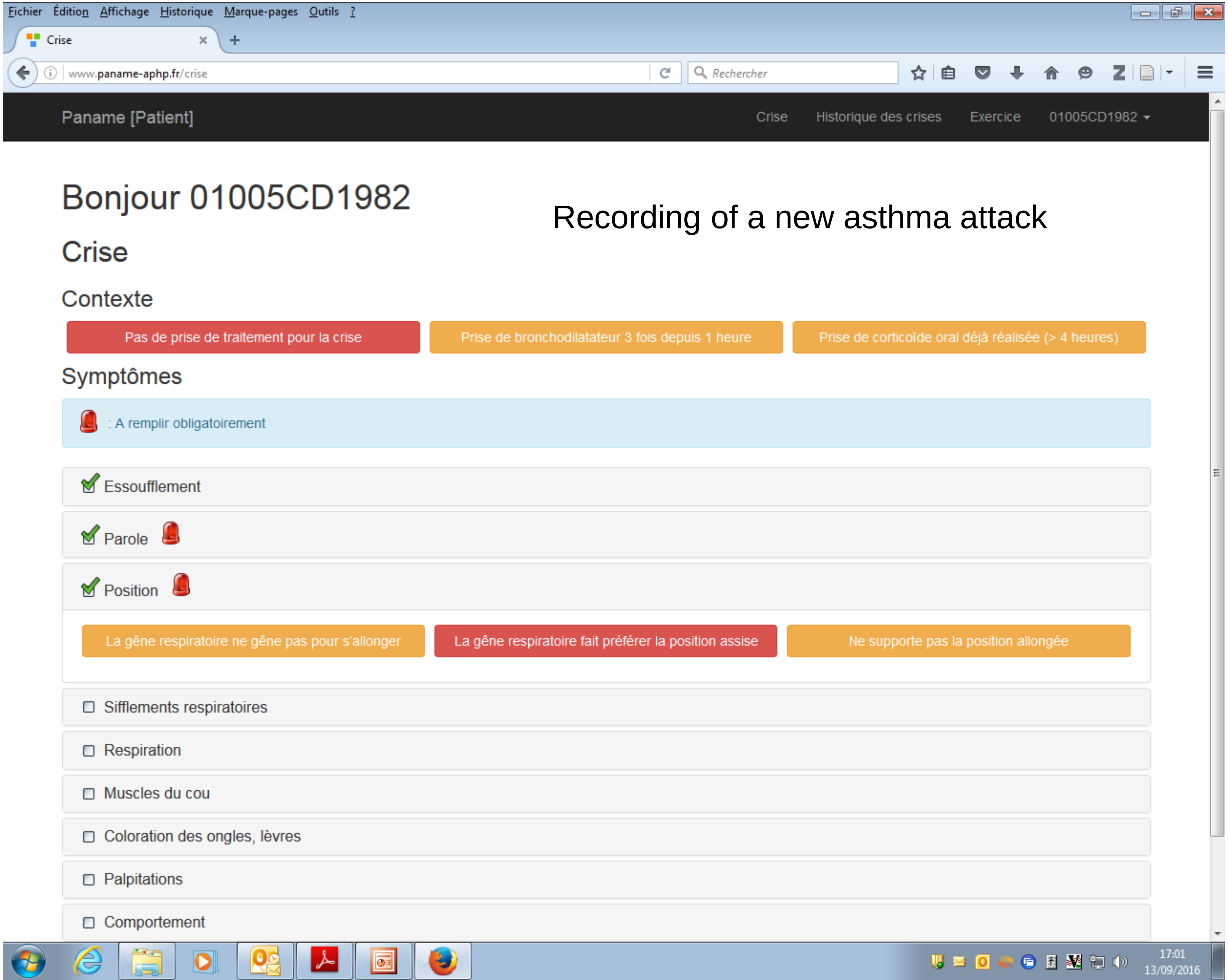

Recording of a new asthma attack

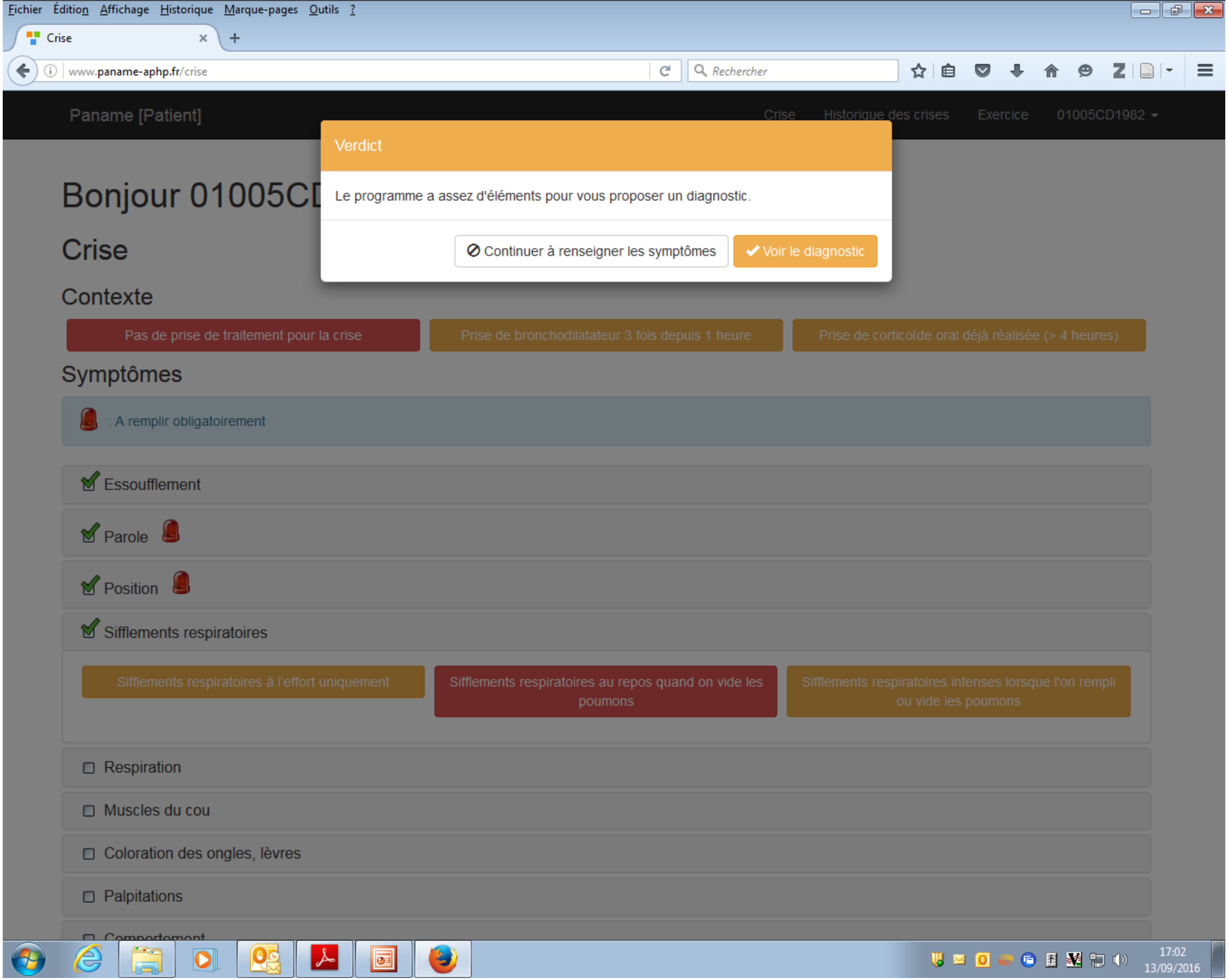
Fichier Édition Affichage Historique Marque-pages Outils ?
Crise
www.paname-aphp.fr/crise
Rechercher
Paname [Patient]
Crise
Historique des crises
Exercice
01005CD1982
Bonjour 01005CD
Crise
Contexte
Pas de prise de traitement pour la crise
Prise de bronchodilatateur 3 fois depuis 1 heure
Prise de corticoïde oral déjà réalisée (> 4 heures)
Symptômes
: A remplir obligatoirement
Essoufflement
Parole
Position
Sifflements respiratoires
Sifflements respiratoires à l'effort uniquement
Sifflements respiratoires au repos quand on vide les poumons
Sifflements respiratoires intenses lorsque l'on rempli ou vide les poumons
Respiration
Muscles du cou
Coloration des ongles, lèvres
Palpitations
Comportement
Verdict
Le programme a assez d'éléments pour vous proposer un diagnostic.
Continuer à renseigner les symptômes
Voir le diagnostic
17:02
13/09/2016

Fichier Édition Affichage Historique Marque-pages Outils ?

Evaluation de la crise

www.paname-aphp.fr/crise/evaluation

Rechercher

Paname [Patient] Crise Historique des crises Exercice 01005CD1982

Bonjour 01005CD1982

Evaluation de la crise

Quel degré de gravité donneriez vous à cette crise, 0 étant le minimum (pas de crise) et 10 le maximum (urgence respiratoire nécessitant le SAMU)?

4

Enregistrer

Paname
Paname :: Crise PAN
Bonjour
Le patient 02001NB1962 a déclaré une crise.

© Paname 2016

Mentions légales

17:02
13/09/2016

Paname [Patient]

Crise | Historique des crises | Exercice | 01005CD1982

Bonjour 01005CD1982

Diagnostic de crise

Diagnostic

Exacerbation modérée non traitée

Prescription

Prendre Ventoline 6 bouffées toutes les 20 minutes 3 fois (=1 heure de traitement). Si amélioration en 1 heure, poursuivre Ventoline 3 bouffées toutes les 4 heures pendant 24 heures puis continuer Ventoline 2 bouffées 4 fois par jour pendant 3 jours puis 3 fois par jour pendant 4 jours. Doubler Innovair pendant 15 jours. Si pas d'amélioration après 1 heure reconnectez vous au programme pour décrire une nouvelle crise en cochant "Prise de bronchodilatateur déjà réalisée".

Diagnosis and treatment for a moderate exacerbation

© Paname 2016

Fichier Édition Affichage Historique Marque-pages Outils ?

Diagnostic de crise

www.paname-aphp.fr/crise/diagnostic

Paname [Patient] Crise Historique des crises Exercice 01005CD1982

Bonjour 01005CD1982

Diagnostic de crise

Diagnostic

Exacerbation grave déjà traitée par bronchodilatateur

Prescription

Appeler le 15 pour prendre un avis médical en urgence et prendre Ventoline 10 bouffées toutes les 20 minutes jusqu'à avis médical et donner Solupred 60 mg (3 comprimés à 20 mg) tout de suite.

Severe exacerbation requiring oral steroid:
Solupred 60 mg (3 comprimés à 20 mg)

© Paname 2016

Mentions légales

17:23 13/09/2016
