# Supplementary material for: Digital Action Plan (Web App) for Managing Asthma Exacerbations: Randomized Controlled Trial
Source: J Med Internet Res. 2023 Jun 29;25:e41490. doi: 10.2196/41490 (PMC10365576; doi:10.2196/41490)
Supplement: Multimedia Appendix 2 [file jmir_v25i1e41490_app2.docx]

### Multimedia Appendix 2

Exacerbation in a child

*For the current symptoms*, bronchodilator taken 3 times in the past hour = no, oral corticosteroids taken for more than 4 hours = no

*Descriptors*, n=5

When the child speaks: Is breathless after saying a whole sentence

Shortness of breath: Shortness of breath when walking

Comfortable position: Can remain lying down

Wheezing: No wheezing or light wheezing

Cough: Coughs a little

*Algorithm diagnosis*: Moderate exacerbation without maximum treatment

*Treatment*:

Give Airomir six puffs every 20 minutes, three times (that is, for one hour).

If improvement after one hour, continue Airomir four puffs every four hours for 24 hours then continue Airomir two puffs four times a day for three days then three times a day for four days.

If no improvement after one hour, reconnect to the program to describe the symptoms after checking “Bronchodilator taken three times in the past hour”.

Exacerbation in an adult

*For the current symptoms*, bronchodilator taken 3 times in the past hour = yes, oral corticosteroids taken for more than 4 hours = no

*Descriptors*, n=5

Speech: Can only say one sentence without taking a breath

Position: Prefers to sit, to decrease respiratory discomfort

Wheezing: Loud wheezing when the lungs fill up or are empty

Breathing rate: increased

Neck muscles: contracted

*Algorithm diagnosis*: Severe exacerbation previously treated with a bronchodilator

*Treatment*:

Call 15 for urgent medical advice and take Bricanyl five puffs every 20 minutes until seen by a healthcare professional and immediately take Cortancyl 40 mg (two 20-mg tablets per day).

NB: Airomir, Bricanyl, and Cortancyl are brand names for the short-acting bronchodilators salbutamol and terbutaline and the oral corticosteroid prednisone, respectively. These names were recorded in the app at participant inclusion by the research team based on the prescription by the physician who included the patient (investigator in charge of the patient). The healthcare emergency telephone number in France is 15.
